# Influence of mutations at different distances from the active center on the activity and stability of laccase 13B22

**DOI:** 10.1186/s40643-025-00893-6

**Published:** 2025-05-27

**Authors:** Ruohan Zhang, Yuchen Wang, Xiaolu Wang, Huiying Luo, Yuan Wang, Bin Yao, Huoqing Huang, Jian Tian, Feifei Guan

**Affiliations:** 1 State Key Laboratory of Animal Nutrition and Feeding, Institute of Animal Sciences, Chinese Academy of Agricultural Sciences, Beijing, 100193 China; 2 National Key Laboratory of Agricultural Microbiology, Biotechnology Research Institute, Chinese Academy of Agricultural Sciences, Beijing, 100081 China

**Keywords:** Laccase, Catalytic activity, Structural distance, Mutant, Thermostability

## Abstract

Laccases with high catalytic efficiency and high thermostability can drive a broader application scope. However, the structural distribution of key amino acids capable of significantly influencing the performance of laccases has not been explored in depth. Thirty laccase 13B22 mutants with changes in amino acids at distances of 5 Å (first shell), 5–8 Å (second shell), and 8–12 Å (third shell) from the active center were validated experimentally with 2,2′-azino-bis(3-ethylbenzothiazoline-6-sulfonic acid) (ABTS) as substrate. Twelve of these mutants (first shell, 1; second shell, 4; third shell, 7) showed higher catalytic efficiency than the wild-type enzyme. Mutants D511E and I88L-D511E showed 5.36- and 10.58-fold increases in *k*_cat_/*K*_m_, respectively, with increases in optimal temperature of 15 °C and optimal pH from 7.0 to 8.0. Furthermore, both mutants exhibited greater thermostability compared to the wild-type, with increases of 3.33 °C and 5.06 °C in *T*_m_ and decreases of 0.39 J and 0.59 J in total structure energy, respectively. The D511E mutation resides in the third shell, while I88L is in the second shell, and their performance enhancements were attributed to alterations in the rigidity or flexibility of specific protein structural domains. Both mutants showed enhanced degradation efficiency for benzo[a]pyrene and zearalenone. These findings highlight the importance of the residues located far from the active center in the function of laccase (second shell and third shell), suggesting broader implications for enzyme optimization and biotechnological applications.

**Supplementary Information:**

The online version contains supplementary material available at 10.1186/s40643-025-00893-6.

## Introduction

The laccases (benzenediol: oxygen oxidoreductase; EC 1.10.3.2) are a group of multicopper-containing oxidoreductases with a wide range of substrates that convert oxygen to water by concomitant four-electron reduction (Janusz et al. 2020). Due to their broad substrate specificity (Chauhan et al. 2017; Agrawal et al. 2019), high redox potential, and green reaction conditions, laccases have been used extensively in industrial processes, including delignification to improve biomass saccharification (Sun et al. 2023), biobleaching (Kumar et al. 2019), and degradation of environmental pollutants or toxic compounds (Singh and Arya 2019; Guan et al. 2018). The varying reaction conditions across different application scenarios impose strict requirements for the catalytic efficiency and stability of laccase (Wiśniewska et al. 2021; Mao et al. 2021).

A number of approaches utilizing protein engineering have been used to enhance the performance of laccases. Typically, in-depth analyses of the three-dimensional (3D) structures of laccases have been performed to identify potential mutation sites and thereby achieve desired properties. For example, Mollania et al. (2011) introduced a single-site E188K mutation in the laccase derived from *Bacillus* sp. HR03, resulting in 3-fold increases in thermal activation and catalytic efficiency (*k*_cat_) compared to the wild-type (WT). Madzak et al. (2005) rationally engineered laccase from the basidiomycete *Pycnoporus cinnabarinus* to enhance its capacity to oxidize phenolic and nonphenolic substrates. After replacing Asp206 (which interacts strongly with the phenolic substrate 2,5-xylidine) with Asn, the *k*_cat_ value of the mutant D206N toward 2,2′-azino-bis(3-ethylbenzothiazoline-6-sulfonic acid) (ABTS) was increased by 3-fold compared to the WT. Copper efflux oxidase (CueO) variants with single, double, and triple mutations in the first and second coordination spheres of the type 1 copper (T1 Cu) active site have been studied (Kataoka et al. 2013). Eleven mutants showed up to 140-fold higher catalytic efficiencies for ABTS than the WT. Most efforts of laccase engineering to date have focused on modifying a specific laccase and cannot be readily applied to others, resulting in low universal efficiency.

Further in-depth research is required to determine universal principles regarding the relations between laccase structure and function. The active site of most laccases is comprised of four Cu atoms located in the T1 site and the trinuclear T2/T3 site. Substrate oxidation occurs at the T1 site, and molecular oxygen is reduced to water at the T2/T3 site. Many studies designed to enhance the enzymatic activity of laccases focused on modifications near the active sites (Ali et al. 2023; Song et al. 2024). However, Gu et al. (2023) suggested the applicability of introducing mutations at distal sites as a flexible/mild form of protein engineering that generally does not cause a radical change in the catalytic center, regulating the overall protein structure and dynamics to achieve synergistic regulation of substrate diversity and multiple functions. Through statistical coupling analysis, Wang et al. (2019) found that the distal site D396 conserved in laccases plays a critical role in proton transfer. Mate et al. (2013) reported that the distance between A461T and T1 Cu in the laccase from the basidiomycete PM1 is 8.86 Å. Meanwhile, A461T established a new network of hydrogen bonds with the mutant S426N, which may change the geometry of the T1 site and may be involved in alternative processing and folding. Furthermore, this structural change facilitated secretion of the laccase in an active, soluble, and stable form. Therefore, further studies are required to determine how amino acids located at different distances from the active center of laccase affect its enzyme activity or properties and whether there are any universal principles. 

Using the laccase 13B22 (Yang et al. 2020), which has been investigated extensively in our laboratory to enhance its soluble expression level and enzymatic activity, we examined the effects of mutations at distances of 5 Å, 5–8 Å, and 8–12 Å from the Cu ion designated as the first, second, and third shells, respectively. By a combination of position-specific amino acid probability (PSAP) analysis and a structure-directed strategy to identify the key sites and mutations affecting its enzyme activity or properties, we successfully obtained two mutants, D511E and I88L-D511E, which exhibited 5.36-fold and 10.58-fold increases in *k*_cat_/*K*_m_ against 2,2′-azino-bis(3-ethylbenzothiazoline-6-sulfonic acid) (ABTS), respectively. Both mutants showed increases of 15 °C at the optimum temperature compared to the WT. Residues 88 and 511 were located in the second shell and third shell, and most of the selected candidate amino acids in the third shell were shown to be effective in improving the catalytic activity. Our work suggested that residues located far from the active center can play pivotal roles in improving catalytic efficiency, providing insights into engineering strategies for laccase and facilitating more effective and precise design of biocatalysts for industrial and environmental applications.

## Materials and methods

### Bacterial strains, plasmids, and chemicals

The bacterial strains and plasmids used in this study were purchased from Tiangen Biotech (Beijing, China). *Escherichia coli* TOP10 and *E. coli* BL21 (DE3) were utilized as the hosts for cloning and gene expression, respectively. The plasmid pET-30a(+)-13B22 harboring the laccase 13B22 gene sequence was synthesized by General Biol (Anhui, China). Chemicals including ABTS, zearalenone (ZEN), and benzo[a]pyrene (BaP) were purchased from Sigma-Aldrich (St. Louis, MO, USA). Kanamycin for strain selection and isopropyl-β-d-1-thiogalactopyranoside (IPTG) for the induction of protein expression were obtained from Solarbio (Beijing, China). Protein purification was performed by Ni-NTA affinity chromatography using a column purchased from Cytiva (Shanghai, China).

### PSAP analysis

The laccase 13B22 was used as a template to search the National Center for Biotechnology Information (NCBI) database (https://www.ncbi.nlm.nih.gov/) for candidate proteins. Identified sequences with > 40% identity and > 50% coverage were downloaded (Meng et al. 2021; Ding et al. 2023). These sequences were aligned with the respective WT and spliced to the same length. Then, PSAP matrices for both enzymes were calculated to determine the conservation of amino acids at each locus (Nguyen et al. 2018). 

### Simulation of protein 3D structure

The three-dimensional structure of laccase 13B22 was predicted from its amino acid sequence using AlphaFold 2.0 with the publicly available code and default parameters (Jumper et al. 2021). Five structures were simulated, and the one exhibiting with highest pLDDT (predicted local distance difference test) and pTM (predicted template modelling) scores was selected for visualization using Pymol. While, AlphaFold2 demonstrates exceptional performance in global structure prediction based on sequence homology, it is not specifically optimized for predicting metal ion coordination. To address this limitation, the active center was obtained through homology modeling using Discovery Studio (2016), with the laccase from *Botrytis aclada* (PDB: 3V9E) as the template (Wang et al. 2021).

### Construction of mutants and site-directed mutagenesis

A recombinant plasmid pET-30a (+)-13B22 harboring the 13B22 coding sequence was used as a template for site-directed two-step PCR mutagenesis (Kirsch and Joly 1998). The primers used for mutagenesis were designed using Oligo ver.7.0 and are listed in Table S3. The PCR products were digested with *Dpn*I (NEB, Ipswich, UK) at 37 °C for 3 h followed by transformation into *E. coli* Top10 competent cells (TransGen, Beijing, China) using standard procedures (Sambrook 2016). The desired mutations were then confirmed by bacterial liquid PCR and sequenced by Tsingke Biological Technology (Beijing, China). The plasmids thus obtained containing the coding sequences of mutant genes were then transfected into *E. coli* BL21 (DE3) cells (TransGen) for protein expression. The beneficial specific single-site mutants served as templates to construct iterative mutants.

### Expression and purification of 13B22

One clone of the resulting recombinant strain, *E. coli* BL21, was picked from Luria-Bertani (LB) plates and cultured in LB medium containing 50 mg/L kanamycin at 37 °C overnight. Then, 1 mL of seed culture was added into 1 L of LB medium containing 50 mg/L kanamycin and cultured at 37 °C for 3 h to an optical density at 600 nm (OD_600_) of 0.6–0.8. Protein expression was induced by adding IPTG and CuCl₂ to the medium to final concentrations of 0.5 mM and 0.25 mM, respectively, followed by incubation at 25 °C with shaking at 120 r/min. Incubation was continued for a further 4 h, during which microaerobic conditions were achieved by switching off the shaking function (Durão et al. 2008). After a further 20 h of growth, cells were harvested, resuspended in 10% Tris-HCl buffer (20 mM, pH 8.0), and lysed by sonication at 4 °C. After centrifugation, the recombinant proteins in the supernatant were purified using Ni-NTA resin according to a previously reported method (Ding et al. 2022). The enzymes were stored at − 80 °C in Tris-HCl buffer (20 mM, pH 8.0) for further use. The purity and molecular mass of 13B22 were assessed by standard sodium dodecyl sulfate-polyacrylamide gel electrophoresis (SDS-PAGE), and gels were stained with Coomassie brilliant blue R250.

### Enzyme activity assay

Laccase activity was investigated according to the method described by Bourbonnais et al. (1995) using ABTS as the substrate. The assay mixture consisted of 200 µL of 5 mM ABTS and 750 µL of 50 mM citrate/phosphate, assayed at 37 °C for 2 min; 50 µL of appropriately diluted laccase protein was then added. After incubation for 3 min, the reaction was stopped by transferring the mixture into an ice-water bath for 30 s. The absorbance was measured at 420 nm (ε = 38 000 M^− 1^ cm^− 1^), and 1 unit of activity (U) was defined as the amount of laccase required to oxidize the substrate to produce 1 µmol of product per minute. Reactions performed with heat-inactivated laccase were used as controls. The kinetic parameters (*K*_m_ and *k*_cat_) of laccases toward ABTS were determined according to the standard laccase assay method (Phillips et al. 2005).

### Effects of temperature and pH on laccase activity and stability

The optimal pH of laccases was determined at 37 °C in buffers with different pH values (4.0–10.0). To evaluate its pH stability, laccases were incubated in buffers of different pH (7.0 and 8.0) at the optimal temperature for 6 h. The effect of temperature on laccase activity was measured in the range of 30–60 °C in 50 mM citrate/phosphate at the respective optimal pH. To assess thermostability, laccases were incubated at 55 °C and 60 °C for various periods. The residual activity was measured according to the standard laccase assay method, and the specific activities were calculated with the enzyme activity at the respective optimal temperatures, with pH values set at 100%, to determine the relative activities at different temperatures and pH values. The melting temperature (*T*_m_) of laccases was measured by differential scanning calorimetry (DSC). Determination was performed in phosphate-buffered saline (PBS, pH 8.0) with purified laccases at a concentration of 0.5 mg/mL, samples were heated from 25 °C to 100 °C, using a cell with a path length of 1 mm. The *T*_m_ was then calculated using Global 3 analysis software.

### Molecular dynamics simulation

The 3D protein structure of 13B22 and mutant molecules, obtained as outlined above, were placed in a water box 12 Å from the edge. Determination of the size and center coordinates of the protein–solvent system, ion neutralization of the solvent environment, and pretreatment of the bound protein and bound solvent were then performed. Molecular dynamics (MD) simulations of proteins at 310 K were performed using NAMD 2.14b2 (Phillips et al. 2005) and CHARMM force fields (MacKerell et al. 1998), including the equilibration and minimization of solvent, energy minimization of ionized proteins, and energy minimization of the protein–solvent system under NPT [constant number (N), pressure (P), and temperature (T)] and NVE [N, volume (V), and energy (E)] conditions in protein dynamic simulations. The simulation was conducted for 250,000,000 steps with a 2 fs step interval, and states were saved at 2500-step intervals, resulting in a total simulation time of 500 ns. Three replicate MD simulations were conducted. The root mean square deviation (RMSD), root mean square fluctuation (RMSF), and radius of gyration were analyzed using MDtraj, and the solvent-accessible surface area (SASA) of the protein was determined with FreeSASA.

### Determination of toxin degradation by laccase

ZEN degradation was performed in a total volume of 400 µL containing 50 mM citrate/phosphate, 0.1 mg/mL ZEN, and 1 mM ABTS with a laccase protein content of 0.05 mg/mL. After 1 h, 1,200 µL of methanol was added to stop the reaction. The degradation of ZEN was analyzed using a high-performance liquid chromatography (HPLC) system (Agilent 1200; Agilent Technologies, Santa Clara, CA, USA) equipped with an Eclipse Plus C18 column (4.6 × 250 mm, 5 μm) with MeOH-H_2_O gradient elution (10–100% for 15 min; 2.0 mL/min), and detection was performed at a wavelength of 316 nm. The primary degradation mixture of BaP contained 50 mM citrate/phosphate, 2 µg/mL BaP, 1 mM ABTS, and 0.25 mg/mL enzymes. After 2 h, 800 µL acetonitrile was added, and the mixture was placed in an ice bath for 1 h to stop the reaction. Then, ultraviolet (UV)-HPLC was performed with an Eclipse Plus C18 column (4.6 × 250 mm, 5 μm) at a detection wavelength of 406 nm to detect BaP with ACN–H_2_O gradient elution (80% for 15 min, 80–100% from 15 to 20 min; 2.0 mL/min). The pH of the solution and reaction temperatures were 7.0/40°C, 7.0/55°C, and 8.0/55°C for 13B22, D511E, and I88L-D511E, respectively.

## Results

### Rational design strategies and screening for 13B22 mutants

**Fig. 1:**
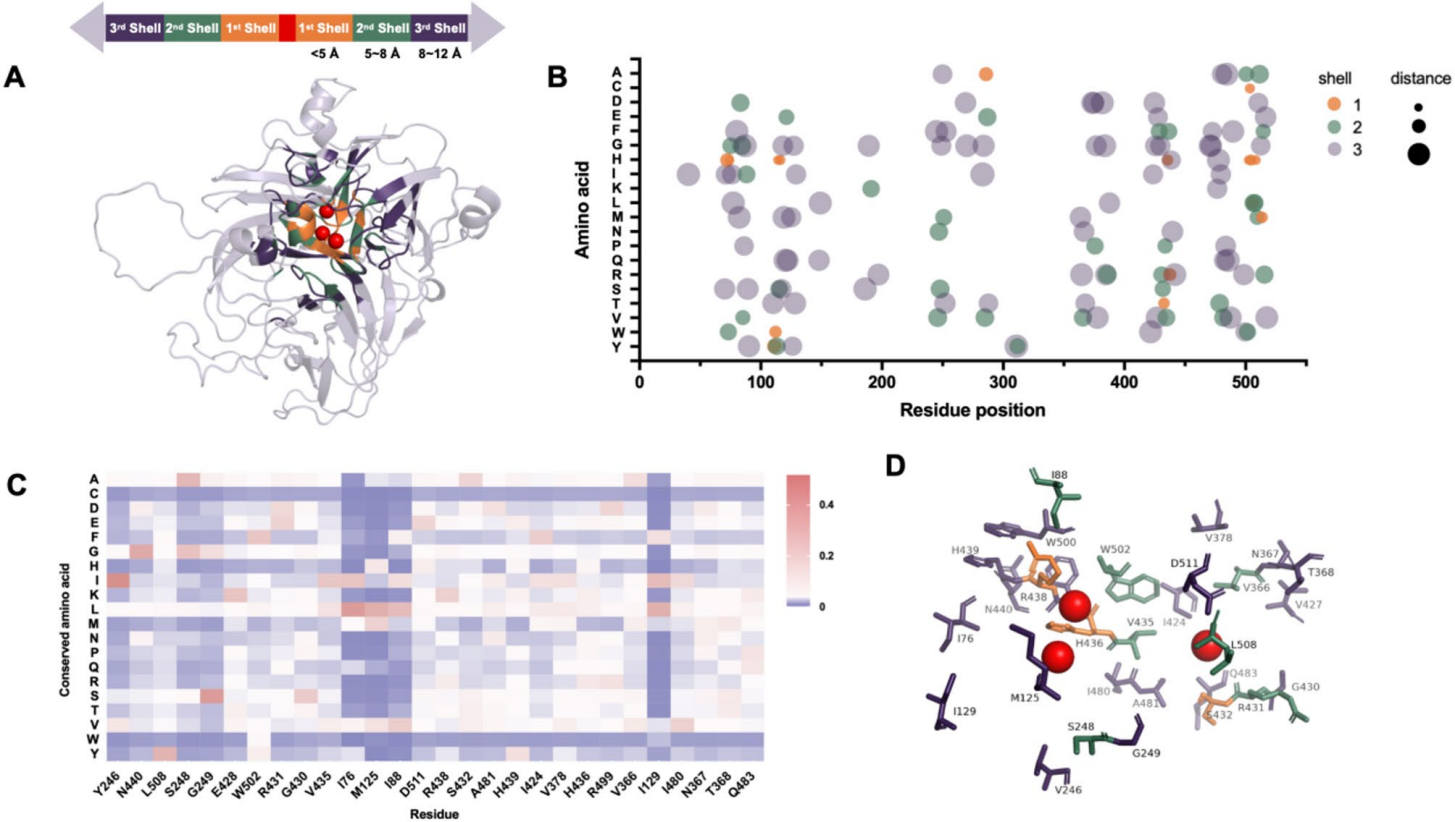
**(A)** The 3D structure and Cu ions of 13B22. Cu atoms are marked in red, and 131 candidate residues with distances to the three Cu ions of 13B22 < 12Å are shown in different colors in the three shells. **(B)** Mutability landscape of 131 candidate residues. The colors of the dots represent the shell. The size of the dots represents the distance to the Cu ions. **(C)** The position-specific amino acid probability (PSAP) of 28 residues. The rows (A to Y) represent one of the 20 amino acids. Each columns represents one position of 28 residues. The colors from blue to red reflect low to high probability at the evolutionary position, respectively. **(D)** Distribution of the 28 mutation sites in the structure

To verify the impacts of amino acids located at different distances from the active site on laccase activity, we chose laccase 13B22 (GenBank accession number: MZ817083) as an example. The laccase 13B22 is valuable in commercial applications and can be used in the oxidative bioremediation of toxic xenobiotic compounds (Yang et al. 2020). Previously, we significantly increased soluble expression levels of laccase 13B22 using the *m*utation *p*redictor for *e*nhanced *p*rotein *e*xpression (MPEPE) deep learning strategy (Ding et al. 2022). To maximize the functions of 13B22, it was necessary to further enhance its stability and catalytic efficiency. Here, the structure of 13B22 was predicted using AlphaFold 2.0 with pLDDT and pTM of 93.2 and 0.919. The active center was homology modeled by Discovery Studio (2016), with the laccase from *Botrytis aclada* (PDB: 3V9E) as the template (Osipov et al. 2014). The results showed that 13B22 contains three Cu atoms per molecule organized into two sites (T1 and T2/T3). The T1 Cu ion is coordinated by one histidine residue (His509) and one cysteine (Cys504) in the equatorial plane and is covered by the side chains of hydrophobic residues in the axial positions. The T2/T3 Cu site includes two T3 Cu ions (Cu3_1_ and Cu3_2_) and eight histidine residues. The main distinguishing feature of the T2/T3 Cu site in the structure of 13B22 is that they do not contain the type 2 Cu ion, i.e., T2 Cu-depleted enzymes (Osipov et al. 2014). Consequently, 131 residues within 12 Å from the active sites were selected and classified into three groups according to their distance from the active site in the protein structure: first shell (< 5Å), second shell (5–8Å), and third shell (8–12Å) (Fig. 1A and Table S1). These residues were evenly distributed across the whole protein sequence (Fig. 1B). The conserved Cu-binding motifs of bacterial laccase, HXHG, HXH, and HCHXXXHXXXXM/L/F, are located in the first shell.

Next, the evolutionary relationships of 1,335 sequences of 13B22 homologous proteins from NCBI were analyzed. Multiple sequence alignment was performed using the PSAP matrix method to calculate the frequency of a specific amino acid occurring at the same position across all sequences (Table S2). Then, the mutation probability values for each amino acid at each position were determined, and the difference value was obtained by subtracting the value of the WT from the value of the residue with the highest mutation probability at this site. A higher difference value indicates that the amino acid is more highly conserved at that position. Based on the results of PSAP, we used a difference value cutoff of > 0.02, and 28 positions were thus obtained. The residues with the highest mutation probability at this site were selected for mutation (Fig. 1C) for experimental validation. H436 and H439 were mutated to R/E and Y/E, respectively. The 28 mutated residues included of 2 in the first shell, 9 in the second shell, and 17 in the third shell (Fig. 1D). Thus, the third shell contained the greatest number of potentially effective mutation sites.

### Enzyme activities of mutants

**Fig. 2:**
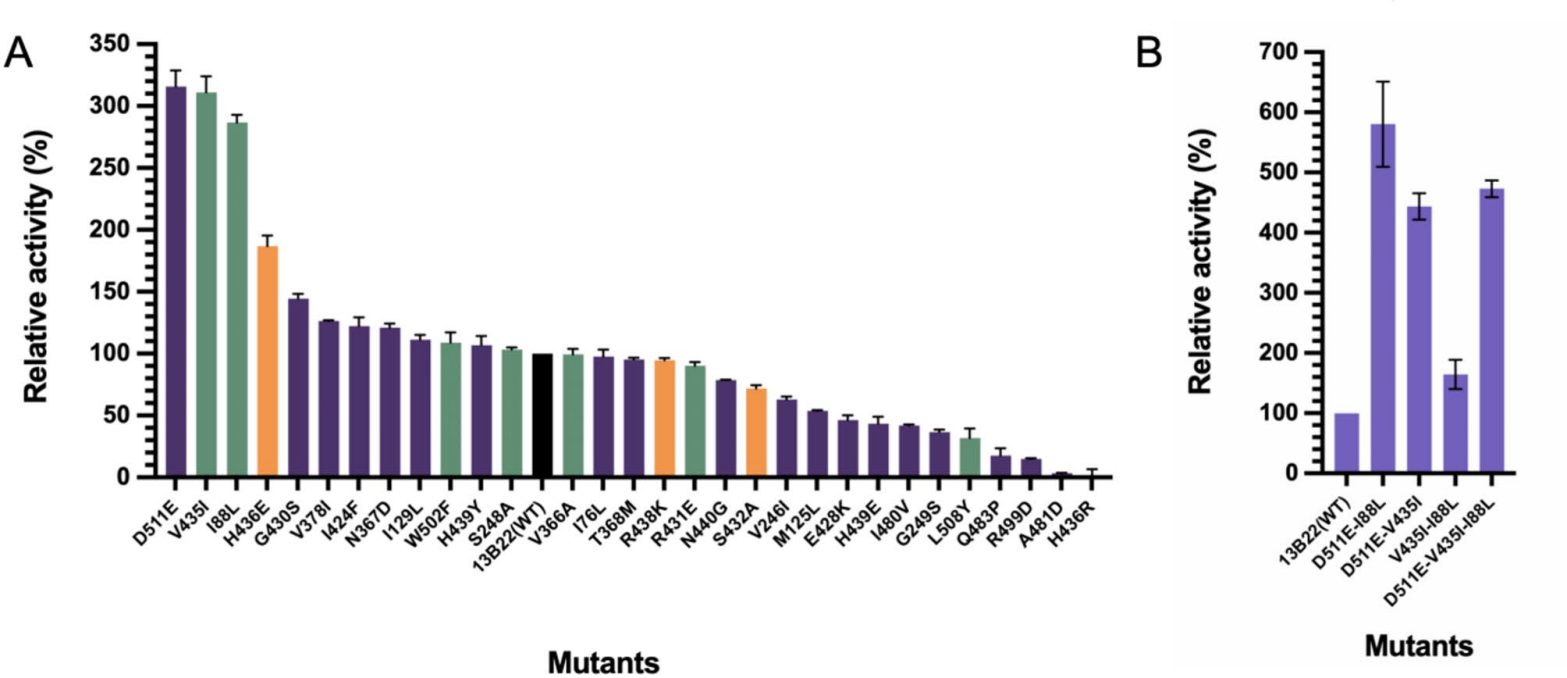
**(A)** Measured enzymatic activity of single-site mutants and WT 13B22. **(B)** Measured enzyme activity of multisite combination mutants and WT 13B22

To validate how the 28 residues in the different shells affect the catalytic activity against ABTS, we introduced 30 individual single-site mutations in laccase 13B22. The proteins of all of the variants were expressed in *E. coli* under the same induction and expression conditions. We characterized their enzymatic activities using laccase activity assays with purified proteins at 40 °C and pH 7.0. Among the 30 mutants, 12 exhibited higher enzymatic activity than the WT (Fig. 2A). Among these 12 mutants, H436 was in the first shell, I88, S248, V435, and W502 were in the second shell, and I129, N367, V378, I424, G430, H439, and D511 were in the third shell. D511E, which was located in the third shell, showed the highest activity, of 811.38 U/g, which was 3.17 times higher than the WT (255.95 U/g). V435I (794.72 U/g) and I88L (732.87 U/g), located in the second shell, also showed activities more than 2.8-fold higher than WT.

To determine whether assembling the beneficial single-site mutations could further enhance the catalytic efficiency, we next generated variants harboring mutations at two and three sites selected based on the magnitude of their effects on activity (Fig. 2A), which were then expressed in *E. coli*. The same enzyme activity assay method used for single-site mutations was also used here. Four generated mutants showed improved catalytic activities (Fig. 2B). Especially, mutant I88L-D511E (1416.19 U/g) showed a 5.82-fold increase in catalytic efficiency compared with the WT, and higher activity than the other mutants I88L-V435I (420.61 U/g), V435I-D511E (1133.86 U/g), and I88L-V435I-D511E (1202.97 U/g).

Comparison of reaction kinetics among 13B22 and the mutants D511E and I88L-D511E (Table 1) showed *k*_cat_/*K*_m_ of 7.424 ± 0.779 mM^− 1^ min^− 1^ for 13B22, while the value for D511E was approximately 5.36-fold higher, at 39.786 ± 0.406 mM^− 1^ min^− 1^. The *k*_cat_/*K*_m_ of I88L-D511E was 78.580 ± 3.615 mM^− 1^ min^− 1^, which was roughly 10.58-fold higher than WT. The *K*_m_ of I88L-D511E (0.541 ± 0.026 mM) was lower than the value for the WT (0.874 ± 0.099 mM) but higher than the value for D511E (0.405 ± 0.011 mM).

**Table 1:** Kinetic parameters of laccase 13B22 and its mutants D511E and I88L-D511E to the substrate of ABTS

Enzyme	K_m_ (mM)	k_cat_ (min^− 1^)	k_cat_/K_m_ (mM^− 1^min^− 1^)
13B22	0.874 ± 0.099	6.413 ± 0.059	7.424 ± 0.779
D511E	0.405 ± 0.011	16.100 ± 0.600	39.786 ± 0.406
I88L-D511E	0.541 ± 0.026	42.440 ± 0.070	78.580 ± 3.615

### Effects of pH and temperature on activity and stability of mutants

**Fig. 3:**
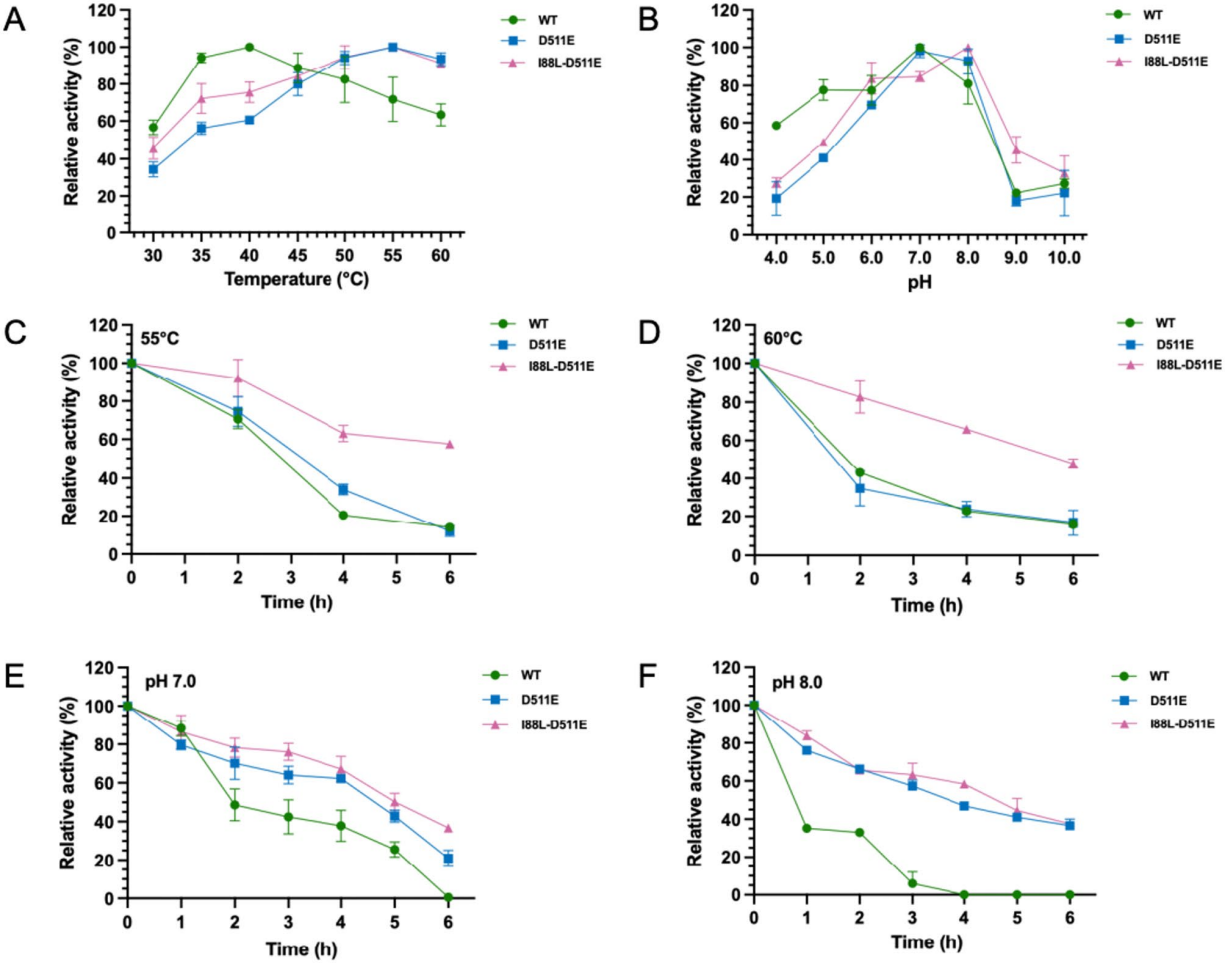
Effects of pH and temperature on the activity and stability of 13B22, D511E, and I88L-D511E. Optimal **(A)** temperature and **(B)** pH for 13B22 and its mutants. (**C** and **D**) Temperature and (**E** and **F**) pH stability of 13B22 and its mutants

To determine the characteristics of I88L-D511E, we measured the optimum conditions for the activity of the mutants against ABTS to assess their potential for industrial applications. In the experiments, we utilized the WT and the optimal single-site mutant D511E for comparison. The optimal temperature for WT was 40 °C, while mutants D511E and I88L-D511E both had an optimal temperature of 55 °C (Fig. 3A). The mutants retained 90% of relative activity at 60 °C, which was 25% higher than WT. The thermostability of I88L-D511E was markedly improved, and it still showed approximately 58% and 48% activity after 6 h of incubation at 55 °C and 60 °C, respectively, while both WT and D511E showed less than 20% activity at these elevated temperatures (Fig. 3C and D).

The optimal pH values of the laccases were then measured at their respective optimal temperatures, and the results indicated that the relative activity of WT and mutants remained above 80% within the pH range of 6.0–8.0 (Fig. 3B). Specifically, WT and D511E showed maximal activity at pH 7.0, while I88L-D511E had an optimal pH of 8.0 (Fig. 3B). WT 13B22 lost more than 50% of its original activity after incubation at pH 7.0 and 8.0 for 2 h (Fig. 3E and F). Compared to the WT, the mutant I88L-D511E retained approximately 40% of its residual enzyme activity after incubation for 6 h (Fig. 3E and F), which suggested increased stability of the enzyme in alkaline solution. In summary, the two-site mutant 13B22 not only exhibited increased enzyme activity but also showed improvements in thermostability and alkaline resistance compared to the WT laccase.

### MD simulation of mutants and WT

To explore the molecular mechanisms underlying the increased thermophilicity and catalytic efficiency of the I88L-D511E two-site mutant, we first examined the *T*_m_ values of the proteins by differential scanning calorimetry. The results showed that D511E and I88L-D511E had *T*_m_ values of 69.40 °C and 71.13 °C, respectively, which were 3.33 °C and 5.06 °C higher than the value for WT 13B22 (66.07 °C) (Table 2). The energy changes (ΔΔ*G*_stability_) of D511E and I88L-D511E calculated using EvoDesign Physical Energy Function (EvoEF) (Pearce et al. 2019) showed that the total structure energy of both mutants decreased by 0.39 J and 0.59 J, respectively. Taken together, these results indicated that D511E and I88L-D511E were more stable than the WT laccase.

**Table 2:** The total structure energy and *T*_m_ value of laccase 13B22 and its mutants D511E and I88L-D511E

Enzyme	T_m_ (℃)	ΔG_stability_ (J)	ΔΔG_stability_ (J)
13B22	66.07	379.14	-
D511E	69.40	378.53	-0.39
I88L-D511E	71.13	378.73	-0.59

**Fig. 4:**
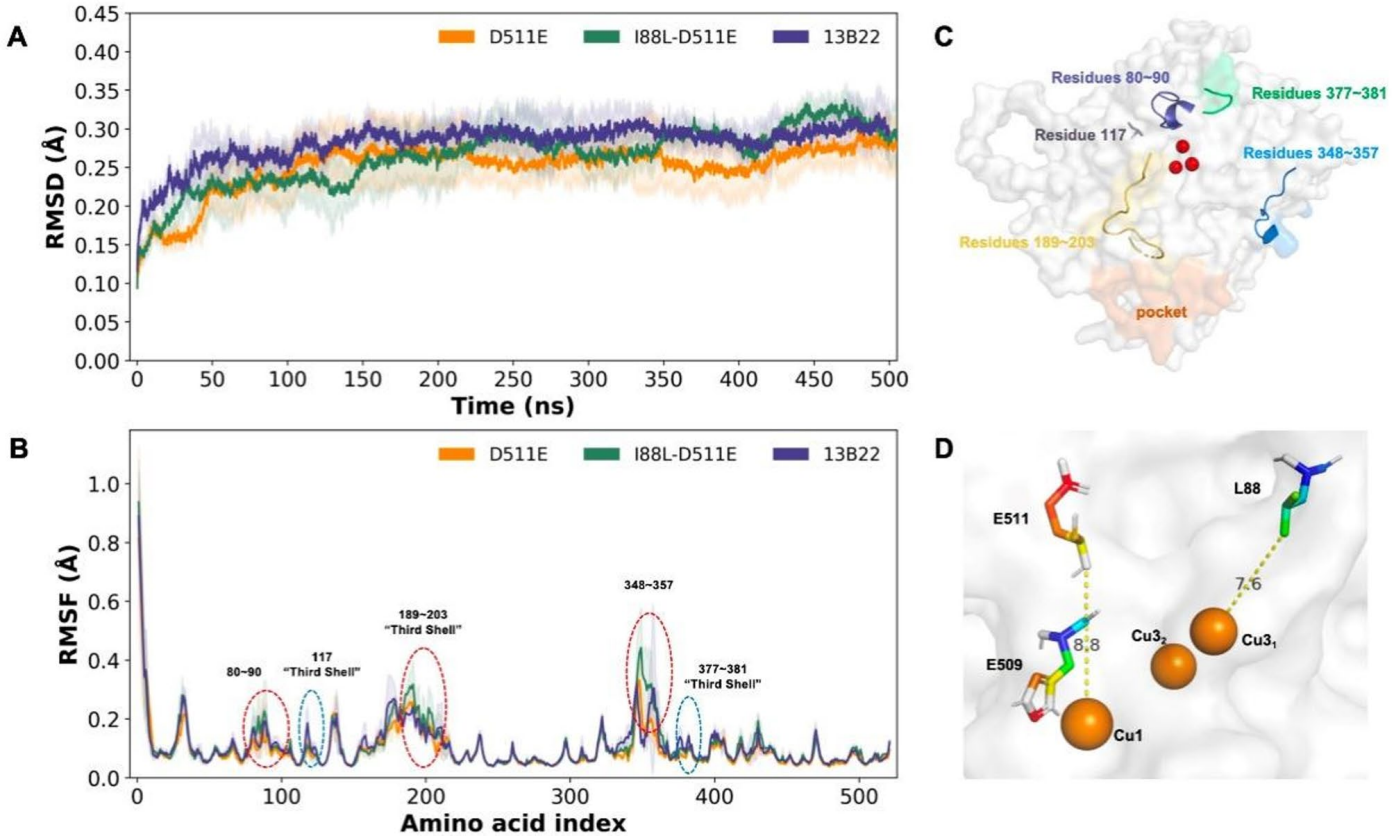
Protein molecular docking, RMSD, and RMSF analyses of molecular dynamics (MD) simulations. **(A)** RMSD and **(B)** RMSF values of the three proteins were calculated by MD simulation using Visual Molecular Dynamics. **(C)** Structural analysis of 13B22. The active site and the secondary structure with increased or decreased fluctuations are indicated. **(D)** Spatial relationship between mutated sites 88 and 511 and the active site

MD simulation analyses showed that the RMSD values were smaller for D511E and I88L-D511E than for WT (Fig. 4A), suggesting that the D511E and I88L mutations effectively increased the overall structural stability of I88L-D511E in the solvent system. The trajectory of RMSF of I88L-D511E showed markedly increased fluctuations in three regions: residues 80–90, 189–203, and 348–357 (Fig. 4B). The 80–90 region, located near the active site, is likely involved in electron transfer (Fig. 4C), as flexibility in this region is crucial for facilitating electron movement during catalysis. Increased flexibility in the 189–203 region, adjacent to the substrate pocket and located in a loop (Fig. 4C), may facilitate substrate binding and conformational changes during catalysis (Chen et al. 2023). Similarly, the 348–357 region is also located in a loop, suggesting that it plays a role in enzyme flexibility and in potential interactions with other molecules. Conversely, the values of RMSF decreased at residues 117 and 377–381 of mutants, which were located in flexible loop regions and close to the active center (Fig. 4C). The increase in stability may help to enhance the overall robustness of the mutants (Pardo et al. 2018).

In terms of structure, Asp511 resides within the third shell, a C-terminal segment located close to the T1 Cu ion within a conserved region (Fig. 4D). The side chain of glutamic acid (Glu) contains an additional methylene group (-CH_2_) compared to Asp511 in the WT. As the T1 Cu ion directly catalyzes substrate oxidation by accepting and transferring electrons, while being coordinated by H509, even minor alterations at Asp511 can potentially influence the electron transfer process (Pearlman et al. 2011). Moreover, position 88 lies only 7.6 Å from one of the T3 Cu ions (Cu3_1_) (Fig. 4D), which has been reported to play a role in the transfer of two electrons and the formation of a peroxide adduct (Mora et al. 2012). Consequently, mutations from isoleucine (I) to leucine (L) at this position may also exert an influence, ultimately enhancing the catalytic activity of the mutant protein.

### Enzymatic degradation of ZEN and BaP

**Fig. 5:**
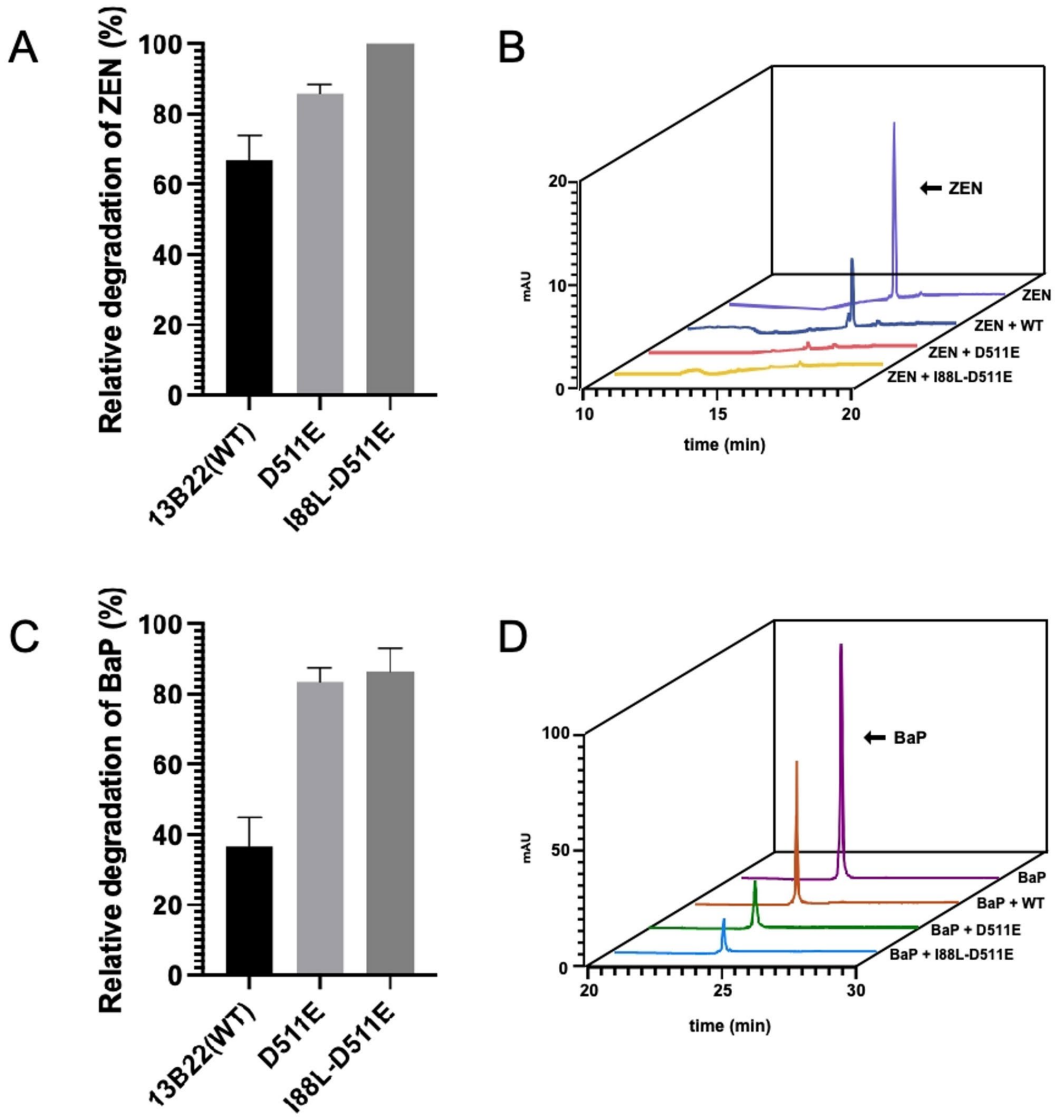
**(A)** Relative degradation of ZEN catalyzed by WT 13B22, D511E, and I88L-D511E. **(B)** HPLC analysis of ZEN degradation with WT 13B22, D511E, and I88L-D511E for 1 h. **(C)** Relative degradation of BaP catalyzed by WT 13B22, D511E, and I88L-D511E. **(D)** HPLC analysis of BaP degradation with WT 13B22, D511E, and I88L-D511E for 2 h

ZEN and BaP are common contaminants in the diet and environment (Pleadin et al. 2019) that have many adverse effects on the ecosystems and on human health (Ropejko and Twarużek 2021; Sun et al. 2024). ZEN is the most common toxic fungal secondary metabolite, and BaP is a high-molecular-weight polycyclic aromatic hydrocarbon produced as a result of incomplete combustion of organic substances. Here, the laccase 13B22 and its mutant derivatives were used to degrade both of these compounds under their respective optimal temperature and pH conditions. A standard curve of ZEN was prepared by HPLC (Figure S1), and degradation rates of ZEN by 13B22 and its mutants were determined. WT 13B22 showed degradation rates of 64%, while the mutants D511E and I88L-D511E showed degradation rates of 85% and 100% after incubation with ZEN for 2 h (Fig. 5A and B). BaP oxidation by 13B22 and its mutants after reaction for 2 h was determined. The degradation of BaP and the formation of oxidation products were analyzed by HPLC. WT 13B22 showed BaP degradation efficiency of 36%, while the values were significantly increased for mutants D511E (81%) and I88L-D511E (85%) (Fig. 5C and D). These results suggested the potential utility of 13B22 for mycotoxin detoxification.

## Discussion

This study was performed to investigate the relationship between amino acid position and laccase function. The results showed that residues located in the second and third shells far from the active center play crucial roles in enhancing laccase catalytic performance. Through site-directed mutagenesis and computational simulations, we successfully optimized the activity of laccase 13B22, yielding significant improvements in catalytic efficiency, optimal temperature, and pH stability. These results provide new insights into the relationship between residue positioning and protein function, highlighting the potential for developing laccase-based applications in industrial and environmental contexts.

The classification of catalytic residues into three concentric shells (5 Å, 5–8 Å and 8–12 Å) integrates both structural observations and functional interaction patterns, while existing literature lacks consensus on shell demarcation distances (Woolery et al. 1984; Tiwari et al. 2014; Anishchenko et al. 2017). Molecular docking revealed a salt bridge (4.9 Å) between substrate ABTS and residue Ala403 in laccase 13B22, establishing 5 Å as the first shell threshold. Previous studies established that the residues that interact directly with substrate also interact with residues in a second shell, and these in turn interact with residues in a third shell (Brodkin et al. 2011). Thus, the distance thresholds (5–8 Å and 8–12 Å) were established. PSAP analysis was performed on 131 sites within a distance of 12 Å from the active center, and more than 60% of the 28 candidate amino acids were located in the third shell. Furthermore, among the 12 mutants with enhanced enzyme activity, 7 of the sites were located in the third shell. Notably, residue 511 located in the third shell contributed significantly to the improvement of both stability and activity of laccase. Similarly, other third shell residues also exhibit similar functional roles, in the laccase derived from *Bacillus licheniformis*, Asp500 (equivalent to Gly513 in 13B22) located in the C-terminal segment and close to the T1 Cu ion (8.6 Å) (Figure S2A and S2B) was shown to play an important role in functional expression (Koschorreck et al. 2009). Luo et al. (2018) reported that residue 501 of laccase CotA, located in the C-terminal segment and 8.5 Å from the active site (Figure S2C), played a significant role in functional expression and contributed to its high thermostability, alkali and salt resistance, and decolorizing efficiency. In the laccase from the *basidiomycete* PM1, A461T located 8.86 Å from the T1 Cu resulted in the establishment of a new network of H-bonds with mutant S426N (8.23 Å from T1 Cu), which could alter the geometry at the T1 site and thus affect the catalytic activity (Mate et al. 2013). These findings highlight the importance of the third shell in the structural and functional characteristics of laccase. They also suggest that this concept could be applicable to other laccases, providing a promising avenue for enzyme optimization and biotechnological applications.

The catalytic efficiency *k*_cat_/*K*_m_ of I88L-D511E mutant (78.580 ± 3.615 mM⁻¹ min⁻¹) was higher than two reported engineered laccase variants: mutant D216N of laccase Lac15 (37.98 ± 10.74 mM^− 1^ min^− 1^) (Xie et al. 2022) and mutant D217K of laccase M17 from *Geobacillus thermocatenulatus* (38.76 × 10^− 4^ mM^− 1^min^− 1^) (Sun et al. 2025). Compared to existing studies, the catalytic efficiency of our mutants demonstrates contextually competitive among the reported values for engineered laccases (Table S4).

The optimal pH for most laccases is 3–5. However, some applications require the optimum to be closer to physiological conditions, such as neutral or even alkaline pH (Yang et al. 2012). I88L-D511E showed a shift in the optimum pH from 7.0 to 8.0 for ABTS, and the two mutants were more stable at pH 8.0. This high alkaline tolerance of I88L-D511E could broaden its application potential, for example making it suitable for organic synthesis, bioremediation (oxidation of pesticides, polyaromatic hydrocarbon (PAH)-containing waste water, dye processing), and pulp biobleaching. Therefore, we examined the degradation capabilities of 13B22 WT and its mutant derivatives against ZEN and BaP. As expected, mutant I88L-D511E showed higher degradation ability against ZEN and BaP than the WT laccase and D511E single-site mutant. The main mechanism of enzyme-induced ZEN degradation involves destroying the lactone structure of ZEN using laccase. The improvement of degradation efficiency was greatly influenced by the pH for I88L-D511E, with an optimum of pH 8, which may explain the observed increase in mycotoxin degradation with increasing pH. The laccase 13B22 is useful not only for toxin degradation but also has wide potential for other industrial and environmental applications.

## Conclusion

Using a PSAP machine learning and structure-guided engineering approach with AlphaFold 2.0 and Discovery Studio to simulate the 3D structure of 13B22, we successfully obtained two mutants derived from WT 13B22, D511E and I88L-D511E, with increased activity (255.95 U/g vs. 811.38 U/g and 1416.19 U/g, respectively). Moreover, these two mutants also exhibited enhanced tolerance to high temperature (55 °C) and pH (8.0). This rational design approach resulted in increases in the degradation capabilities from 36 to 85% for ZEN and from 64 to 100% for BaP. Taken together, these results suggested that these two mutant laccases have industrial and environmental application potential.

## Electronic supplementary material

Below is the link to the electronic supplementary material.

Supplementary Material 1

Supplementary Material 2

Supplementary Material 3

## Data Availability

The datasets used and/or analyzed during the current study are available from the corresponding author on reasonable request.
