# Supplementary material for: Influence of mutations at different distances from the active center on the activity and stability of laccase 13B22
Source: Bioresour Bioprocess. 2025 May 27;12(1):47. doi: 10.1186/s40643-025-00893-6 (PMC12116972; doi:10.1186/s40643-025-00893-6)
Supplement: Supplementary file 1 — Supplementary Material 1 [file 40643_2025_893_MOESM1_ESM.pdf]

Table S2 The position-specific amino acid probability (PSAP) of 13B22

No	AA	A	C	D	E	F	G	H	I	K	L	M	N	P	Q	R	S	T	V	W	Y	Entropy	Wt	Max	Max_aa	Diff
1	A	0.1066	0.0189	0.0494	0.0595	0.038	0.0697	0.022	0.0511	0.0565	0.0873	0.023	0.0408	0.0483	0.0393	0.0491	0.0764	0.0565	0.0636	0.0133	0.0305	0.9642	0.1066	0.1066	A	0
2	D	0.0762	0.0187	0.0561	0.0697	0.0407	0.0714	0.0218	0.0507	0.056	0.0866	0.0228	0.0419	0.0491	0.0529	0.0509	0.072	0.056	0.0631	0.0132	0.0303	0.9703	0.0561	0.0866	L	0.0305
3	A	0.1146	0.0185	0.0485	0.0584	0.0373	0.0684	0.0216	0.0502	0.0554	0.0857	0.0226	0.0401	0.0474	0.0386	0.0482	0.0804	0.0555	0.0624	0.0131	0.033	0.9618	0.1146	0.1146	A	0
4	V	0.0931	0.0185	0.0485	0.0584	0.0386	0.0684	0.0216	0.0502	0.0554	0.0907	0.0226	0.0401	0.052	0.0521	0.0482	0.0643	0.0585	0.0713	0.0175	0.03	0.969	0.0713	0.0931	A	0.0218
5	D	0.0761	0.0177	0.0834	0.0644	0.0356	0.0775	0.0206	0.0479	0.0575	0.0818	0.0216	0.0531	0.049	0.043	0.046	0.0678	0.0563	0.0596	0.0125	0.0286	0.9664	0.0834	0.0834	D	0
6	T	0.0729	0.0165	0.043	0.0519	0.0331	0.0651	0.0192	0.0446	0.0531	0.0794	0.0201	0.0356	0.042	0.0652	0.0475	0.0588	0.157	0.0568	0.0116	0.0266	0.9451	0.157	0.157	T	0
7	T	0.0776	0.0128	0.0334	0.0404	0.0258	0.0526	0.0204	0.051	0.0384	0.065	0.0157	0.0588	0.0353	0.0308	0.0335	0.0927	0.2267	0.0573	0.009	0.0229	0.9015	0.2267	0.2267	T	0
8	T	0.0642	0.0127	0.0337	0.0406	0.0364	0.0601	0.0149	0.0391	0.0381	0.0747	0.0155	0.0362	0.0553	0.0266	0.0391	0.2313	0.0922	0.06	0.0089	0.0206	0.898	0.0922	0.2313	S	0.1391
9	D	0.0612	0.0117	0.0935	0.0386	0.0234	0.1177	0.0519	0.031	0.0353	0.0578	0.0141	0.0337	0.2047	0.0293	0.0305	0.0521	0.0481	0.0387	0.0082	0.0188	0.9019	0.0935	0.2047	P	0.1112
10	G	0.0397	0.0095	0.0329	0.0404	0.0189	0.3994	0.0149	0.025	0.0613	0.043	0.0162	0.0281	0.028	0.0456	0.027	0.0483	0.0647	0.0312	0.0066	0.0193	0.789	0.3994	0.3994	G	0
11	A	0.1898	0.0084	0.0253	0.0705	0.0182	0.0375	0.0161	0.0233	0.0254	0.0757	0.0128	0.0204	0.051	0.2215	0.033	0.0462	0.029	0.0762	0.0059	0.0137	0.8472	0.1898	0.2215	Q	0.0317
12	P	0.0577	0.0069	0.021	0.0386	0.0138	0.035	0.0087	0.0184	0.0214	0.0316	0.0083	0.0397	0.532	0.0199	0.0284	0.0337	0.0459	0.023	0.0048	0.0111	0.664	0.532	0.532	P	0
13	V	0.0787	0.0047	0.0124	0.0701	0.0097	0.0223	0.011	0.0973	0.0145	0.1097	0.014	0.0103	0.012	0.0172	0.0148	0.0441	0.0398	0.4061	0.0034	0.0078	0.7202	0.4061	0.4061	V	0
14	L	0.0076	0.0011	0.0028	0.0033	0.0039	0.004	0.0012	0.0074	0.0032	0.92	0.004	0.0023	0.0032	0.0022	0.0028	0.0037	0.0032	0.0216	0.0008	0.0018	0.1622	0.92	0.92	L	0
15	S	0.2126	0.0011	0.0064	0.0176	0.0022	0.0129	0.0017	0.0029	0.0187	0.0062	0.0013	0.0064	0.004	0.0336	0.0469	0.4	0.2184	0.0045	0.0008	0.0018	0.5688	0.4	0.4	S	0
16	G	0.0044	0.001	0.0045	0.0066	0.002	0.9443	0.0013	0.0025	0.0032	0.0044	0.0012	0.0028	0.0026	0.0022	0.0035	0.0045	0.003	0.0037	0.0007	0.0016	0.1241	0.9443	0.9443	G	0
17	T	0.0058	0.001	0.0962	0.0127	0.0018	0.0035	0.0029	0.004	0.072	0.0042	0.0011	0.1163	0.0239	0.0341	0.034	0.1017	0.4795	0.0032	0.0006	0.0015	0.5991	0.4795	0.4795	T	0
18	E	0.0203	0.0009	0.2102	0.4684	0.0018	0.0053	0.0269	0.0122	0.0042	0.0043	0.0012	0.0211	0.0026	0.0666	0.0078	0.0775	0.0369	0.0296	0.0007	0.0015	0.59	0.4684	0.4684	E	0
19	F	0.0058	0.001	0.0025	0.0029	0.8707	0.0035	0.0014	0.049	0.0028	0.0164	0.0107	0.0021	0.0024	0.002	0.0025	0.0033	0.0028	0.0117	0.0008	0.0056	0.2255	0.8707	0.8707	F	0
20	D	0.0763	0.0009	0.6827	0.087	0.0147	0.0039	0.0348	0.0024	0.0029	0.0072	0.0011	0.0443	0.0024	0.0142	0.005	0.0078	0.0044	0.0039	0.0006	0.0035	0.4332	0.6827	0.6827	D	0
21	L	0.0036	0.0009	0.0023	0.0028	0.0021	0.0033	0.001	0.0167	0.0026	0.9417	0.0014	0.0019	0.0023	0.0018	0.0023	0.0031	0.0027	0.0053	0.0007	0.0015	0.1245	0.9417	0.9417	L	0
22	T	0.0276	0.001	0.0718	0.0496	0.3006	0.0036	0.0115	0.0059	0.0029	0.0304	0.0037	0.0271	0.0024	0.0041	0.0133	0.0704	0.1462	0.0671	0.0032	0.1575	0.7351	0.1462	0.3006	F	0.1545
23	I	0.0036	0.0009	0.0023	0.0027	0.002	0.0032	0.001	0.8062	0.0026	0.0182	0.0012	0.0019	0.0022	0.0018	0.0022	0.003	0.0027	0.1399	0.0006	0.0015	0.2465	0.8062	0.8062	I	0
24	A	0.0859	0.0009	0.1405	0.0166	0.0019	0.6007	0.0012	0.0039	0.0031	0.0041	0.0011	0.0035	0.0025	0.0021	0.025	0.0801	0.0067	0.0181	0.0006	0.0015	0.4738	0.0859	0.6007	G	0.5148
25	E	0.0203	0.0009	0.0097	0.624	0.0018	0.0137	0.0012	0.0024	0.0901	0.0042	0.0011	0.0086	0.0104	0.1049	0.0196	0.0388	0.0203	0.0031	0.0006	0.0241	0.4957	0.624	0.624	E	0
26	T	0.0519	0.001	0.0025	0.0028	0.0018	0.0034	0.0011	0.0242	0.0484	0.0674	0.037	0.0073	0.0023	0.0753	0.0043	0.099	0.5507	0.0176	0.0006	0.0015	0.5611	0.5507	0.5507	T	0
27	P	0.0782	0.0009	0.0122	0.0039	0.0019	0.0175	0.0011	0.0026	0.0079	0.0195	0.0281	0.003	0.6136	0.0822	0.0092	0.082	0.0268	0.0072	0.0007	0.0015	0.5075	0.6136	0.6136	P	0
28	V	0.0348	0.0009	0.0022	0.0027	0.0122	0.0032	0.001	0.0321	0.0026	0.019	0.0139	0.0019	0.0022	0.0018	0.0022	0.003	0.0027	0.8591	0.0006	0.0015	0.2411	0.8591	0.8591	V	0
29	N	0.0034	0.0009	0.0602	0.0027	0.0018	0.0034	0.0012	0.049	0.0027	0.004	0.0011	0.7975	0.0022	0.0018	0.011	0.0077	0.0401	0.0071	0.0006	0.0015	0.305	0.7975	0.7975	N	0
30	F	0.0043	0.0011	0.0023	0.0028	0.3115	0.0036	0.0011	0.3235	0.0026	0.1753	0.0024	0.002	0.0025	0.0019	0.0024	0.0034	0.0031	0.0839	0.0008	0.0695	0.5484	0.3115	0.3235	I	0.012
31	T	0.0068	0.0009	0.0163	0.0028	0.0018	0.0091	0.0011	0.0024	0.0027	0.004	0.0011	0.0759	0.0023	0.0019	0.0023	0.108	0.7556	0.0031	0.0006	0.0014	0.3219	0.7556	0.7556	T	0
32	G	0.0046	0.0009	0.0045	0.0116	0.0018	0.9437	0.0012	0.0023	0.0029	0.0039	0.0011	0.0026	0.0024	0.002	0.0025	0.0045	0.0027	0.0029	0.0006	0.0014	0.1231	0.9437	0.9437	G	0
33	A	0.1036	0.0009	0.0025	0.0155	0.0017	0.0033	0.0104	0.0037	0.2048	0.007	0.0011	0.1191	0.0023	0.036	0.1737	0.1837	0.082	0.0466	0.0006	0.0015	0.7177	0.1036	0.2048	K	0.1012
34	P	0.2088	0.0009	0.0185	0.0378	0.0018	0.0034	0.0066	0.0065	0.0448	0.0074	0.016	0.0171	0.3203	0.0276	0.0452	0.086	0.1085	0.0404	0.0006	0.0014	0.714	0.3203	0.3203	P	0
35	R	0.1042	0.0047	0.0025	0.0034	0.0073	0.0271	0.0043	0.0023	0.0308	0.0045	0.0078	0.0022	0.0022	0.0024	0.6835	0.09	0.0109	0.0078	0.0006	0.0014	0.419	0.6835	0.6835	R	0
36	M	0.0047	0.0009	0.0047	0.0027	0.0017	0.0032	0.0044	0.0663	0.0201	0.0464	0.1319	0.0105	0.0375	0.0376	0.0339	0.0313	0.5192	0.0257	0.0037	0.0136	0.6092	0.1319	0.5192	T	0.3873
37	A	0.9214	0.0011	0.0022	0.0026	0.0018	0.0311	0.001	0.0026	0.0025	0.0043	0.0012	0.0018	0.0021	0.0018	0.0022	0.0036	0.0112	0.0036	0.0006	0.0014	0.1512	0.9214	0.9214	A	0
38	T	0.0037	0.001	0.0022	0.0026	0.0022	0.0032	0.0026	0.0494	0.0504	0.103	0.3311	0.0019	0.0022	0.0591	0.0022	0.0034	0.308	0.0696	0.0007	0.0016	0.5981	0.308	0.3311	M	0.0231
39	T	0.1005	0.0009	0.0022	0.0025	0.0016	0.0154	0.0009	0.0021	0.0024	0.0857	0.001	0.0019	0.0021	0.0017	0.0021	0.0127	0.7391	0.0236	0.0005	0.0013	0.3402	0.7391	0.7391	T	0
40	I	0.0121	0.0005	0.0011	0.0013	0.0011	0.0016	0.0005	0.6859	0.0013	0.0095	0.0218	0.0009	0.0011	0.0009	0.0011	0.0015	0.0014	0.2555	0.0003	0.0008	0.2979	0.6859	0.6859	I	0
41	N	0.0017	0.0005	0.0108	0.0013	0.0009	0.0018	0.0007	0.0011	0.0014	0.0019	0.0005	0.9682	0.0011	0.0009	0.0012	0.002	0.0015	0.0014	0.0003	0.0007	0.0734	0.9682	0.9682	N	0
42	G	0.0205	0.0005	0.0793	0.0017	0.0009	0.8443	0.0057	0.0012	0.0017	0.0021	0.0006	0.0269	0.0013	0.0011	0.0036	0.0045	0.0015	0.0015	0.0003	0.0008	0.2323	0.8443	0.8443	G	0
43	S	0.0061	0.0005	0.0013	0.0014	0.0009	0.2866	0.0005	0.0049	0.0014	0.0063	0.0205	0.0017	0.0012	0.0279	0.0024	0.5712	0.0586	0.0055	0.0003	0.0007	0.4085	0.5712	0.5712	S	0
44	L	0.0018	0.0005	0.0011	0.0013	0.0012	0.0015	0.0005	0.3394	0.0012	0.5299	0.0121	0.0009	0.0011	0.0009	0.0011	0.0014	0.0132	0.0901	0.0003	0.0007	0.3788	0.5299	0.5299	L	0
45	P	0.013	0.0005	0.0015	0.0018	0.001	0.0019	0.0006	0.0013	0.0017	0.0023	0.0006	0.0011	0.9602	0.0011	0.0014	0.0057	0.0016	0.0018	0.0003	0.0008	0.0882	0.9602	0.9602	P	0
46	A	0.2435	0.0005	0.0018	0.0017	0.0009	0.7286	0.0007	0.0012	0.0017	0.002	0.0006	0.0017	0.0013	0.0011	0.0014	0.007	0.0015	0.0015	0.0003	0.0008	0.2495	0.2435	0.7286	G	0.485
47	P	0.0016	0.0004	0.0011	0.0013	0.0008	0.0014	0.0006	0.001	0.0012	0.0025	0.0005	0.0009	0.9793	0.0009	0.0011	0.0014	0.0018	0.0013	0.0003	0.0007	0.0532	0.9793	0.9793	P	0
48	T	0.0016	0.0004	0.001	0.0012	0.0011	0.0014	0.0005	0.1066	0.0011	0.4318	0.0014	0.0008	0.001	0.0125	0.001	0.0013	0.3661	0.0681	0.0003	0.0007	0.4368	0.3661	0.4318	L	0.0658
49	L	0.0016	0.0004	0.001	0.0012	0.0011	0.0014	0.0005	0.0145	0.0011	0.9669	0.0014	0.0008	0.001	0.0008	0.001	0.0013	0.0012	0.0017	0.0003	0.0007	0.0735	0.9669	0.9669	L	0
50	R	0.0015	0.0004	0.001	0.0013	0.0008	0.0014	0.0205	0.001	0.096	0.0018	0.0005	0.0009	0.001	0.0009	0.8451	0.0014	0.0012	0.0192	0.0003	0.0039	0.2164	0.8451	0.8451	R	0
51	W	0.0015	0.0063	0.001	0.0013	0.0541	0.0014	0.0006	0.001	0.0012	0.0514	0.0957	0.0009	0.001	0.0009	0.0011	0.0014	0.0012	0.0013	0.7763	0.0007	0.2917	0.7763	0.7763	W	0
52	R	0.002	0.0004	0.0013	0.0018	0.0008	0.0016	0.005	0.0011	0.2147	0.002	0.0006	0.0058	0.0011	0.0391	0.7051	0.0017	0.0021	0.0127	0.0003	0.0007	0.3107	0.7051	0.7051	R	0
53	E	0.0015	0.0004	0.001	0.9509	0.0008	0.0014	0.0006	0.001	0.0012	0.0018	0.0005	0.0009	0.001	0.0283	0.0011	0.0014	0.0012	0.0044	0.0003	0.0006	0.0953	0.9509	0.9509	E	0
54	G	0.0022	0.0004	0.0023	0.0016	0.0009	0.975	0.0006	0.0011	0.0016	0.0019	0.0005	0.0016	0.0012	0.001	0.0013	0.0028	0.0014	0.0014	0.0003	0.0007	0.0626	0.975	0.975	G	0
55	D	0.0017	0.0004	0.8027	0.0709	0.0008	0.0033	0.0006	0.001	0.0014	0.0018	0.0005	0.0018	0.0011	0.0129	0.0011	0.0298	0.0654	0.0013	0.0003	0.0011	0.2747	0.8027	0.8027	D	0
56	T	0.0047	0.0005	0.0503	0.0483	0.0008	0.0016	0.0005	0.0067	0.0078	0.0019	0.0188	0.062	0.0045	0.003	0.0011	0.0287	0.7516	0.0065	0.0003	0.0007	0.3608	0.7516	0.7516	T	0
57	V	0.0107	0.0004	0.001	0.0012	0.001	0.0014	0.0005	0.0807	0.0011	0.0084	0.0006	0.0008	0.001	0.0008	0.001	0.0013	0.0012	0.8858	0.0003	0.0007	0.1668	0.8858	0.8858	V	0
58	T	0.0179	0.0005	0.0027	0.0064	0.0008	0.0016	0.0045	0.012	0.0017	0.0019	0.0005	0.032	0.0011	0.0009	0.0032	0.0396	0.8263	0.0455	0.0003	0.0007	0.2735	0.8263	0.8263	T	0
59	I	0.0016	0.0004	0.001	0.0012	0.0011	0.0014	0.0004	0.2597	0.0011	0.7	0.0135	0.0008	0.001	0.0008	0.001	0.0013	0.0012	0.0114	0.0003	0.0007	0.2721	0.2597	0.7	L	0.4402
60	R	0.0205	0.0003	0.0009	0.001	0.0021	0.0012	0.0402	0.0008	0.0354	0.0043	0.0004	0.0416	0.0008	0.003	0.8285	0.0011	0.001	0.001	0.0002	0.0154	0.2656	0.8285	0.8285	R	0
61	V	0.0018	0.0003	0.0008	0.001	0.0006	0.0012	0.0005	0.007	0.0009	0.014	0.0004	0.0008	0.0008	0.0008	0.0009	0.0011	0.0009	0.9654	0.0002	0.0005	0.0747	0.9654	0.9654	V	0
62	T	0.0156	0.0004	0.0073	0.0017	0.0007	0.0014	0.1093	0.001	0.2136	0.0017	0.0005	0.0035	0.0009	0.0027	0.3322	0.0361	0.2693	0.0013	0.0002	0.0006	0.5403	0.2693	0.3322	R	0.0629
63	N	0.0013	0.0004	0.0012	0.001	0.0007	0.0014	0.0006	0.0009	0.001	0.0015	0.0004	0.9828	0.0008	0.0007	0.0009	0.0015	0.0011	0.0011	0.0002	0.0006	0.0455	0.9828	0.9828	N	0
64	R	0.0272	0.0004	0.0039	0.0092	0.0007	0.0043	0.0933	0.001	0.0466	0.0094	0.0005	0.0193	0.001	0.104	0.6517	0.0016	0.0228	0.0024	0.0002	0.0006	0.4432	0.6517	0.6517	R	0
65	L	0.0013	0.0003	0.0008	0.001	0.0008	0.0011	0.0004	0.0012	0.0009	0.8836	0.1013	0.0007	0.0008	0.0007	0.0008	0.0011	0.001	0.0012	0.0002	0.0005	0.1493	0.8836	0.8836	L	0
66	P	0.1529	0.0004	0.1811	0.0159	0.0008	0.0185	0.0047	0.001	0.1827	0.0018	0.0005	0.1148	0.0938	0.038	0.0762	0.086	0.0282	0.0018	0.0003	0.0007	0.7424	0.0938	0.1827	K	0.0889
67	V	0.051	0.0003	0.0866	0.522	0.0007	0.0043	0.021	0.001	0.0179	0.0038	0.0019	0.0016	0.0195	0.0839	0.019	0.0018	0.0342	0.1287	0.0003	0.0006	0.5656	0.1287	0.522	E	0.3933
68	D	0.0708	0.0004	0.2828	0.0181	0.0007	0.0101	0.0043	0.0017	0.0012	0.0033	0.0142	0.0276	0.2313	0.0281	0.001	0.1582	0.123	0.0156	0.0048	0.0029	0.6661	0.2828	0.2828	D	0
69	S	0.0048	0.0004	0.001	0.0011	0.0007	0.0044	0.0004	0.001	0.0011	0.0015	0.0004	0.001	0.0009	0.0007	0.0009	0.1234	0.8541	0.0013	0.0002	0.0006	0.1787	0.1234	0.8541	T	0.7307
70	S	0.0085	0.0003	0.0009	0.001	0.0006	0.0012	0.0005	0.0008	0.001	0.0014	0.0004	0.0008	0.0008	0.0008	0.0009	0.967	0.0114	0.0011	0.0002	0.0005	0.0725	0.967	0.967	S	0
71	I	0.0012	0.0003	0.0008	0.001	0.0006	0.0012	0.0005	0.9464	0.0009	0.0316	0.0004	0.0008	0.0008	0.0008	0.0009	0.0011	0.0009	0.0089	0.0002	0.0005	0.0988	0.9464	0.9464	I	0
72	H	0.0012	0.0003	0.0008	0.001	0.0006	0.0012	0.9842	0.0008	0.0009	0.0014	0.0004	0.0008	0.0008	0.0008	0.0009	0.0011	0.0009	0.001	0.0002	0.0005	0.0423	0.9842	0.9842	H	0
73	W	0.0012	0.0003	0.0008	0.001	0.0006	0.0012	0.0005	0.0008	0.0009	0.0014	0.0004	0.0008	0.0008	0.0008	0.0009	0.0011	0.0009	0.001	0.9839	0.0005	0.0431	0.9839	0.9839	W	0
74	H	0.0012	0.0003	0.0008	0.001	0.0006	0.0012	0.9842	0.0008	0.0009	0.0014	0.0004	0.0008	0.0008	0.0008	0.0009	0.0011	0.0009	0.001	0.0002	0.0005	0.0423	0.9842	0.9842	H	0
75	G	0.0014	0.0003	0.001	0.0011	0.0006	0.9836	0.0005	0.0008	0.001	0.0015	0.0004	0.0009	0.0009	0.0008	0.0009	0.0013	0.001	0.0011	0.0002	0.0006	0.0435	0.9836	0.9836	G	0
76	I	0.0014	0.0004	0.0008	0.001	0.0098	0.0011	0.0004	0.7843	0.0009	0.1615	0.0293	0.0007	0.0008	0.0007	0.0008	0.0011	0.001	0.0034	0.0003	0.0006	0.2457	0.7843	0.7843	I	0
77	L	0.0014	0.0004	0.0008	0.001	0.001	0.0011	0.0004	0.4872	0.0009	0.443	0.0012	0.0007	0.0008	0.0007	0.0195	0.0011	0.001	0.0373	0.0003	0.0006	0.3346	0.443	0.4872	I	0.0442
78	L	0.0013	0.0003	0.0008	0.001	0.0009	0.0011	0.0004	0.0013	0.0009	0.8443	0.0006	0.0007	0.0038	0.0007	0.0008	0.0011	0.0077	0.1313	0.0002	0.0006	0.1866	0.8443	0.8443	L	0
79	P	0.0013	0.0003	0.0111	0.001	0.0006	0.0012	0.0005	0.0008	0.001	0.0015	0.0004	0.0008	0.9741	0.0008	0.0009	0.0011	0.001	0.001	0.0002	0.0005	0.0599	0.9741	0.9741	P	0
80	F	0.5897	0.0006	0.0009	0.0067	0.1977	0.0017	0.0043	0.0012	0.001	0.002	0.0006	0.0008	0.0106	0.0008	0.0009	0.0746	0.0276	0.0057	0.0003	0.0724	0.4415	0.1977	0.5897	A	0.3921
81	Q	0.0082	0.0004	0.0497	0.1247	0.0007	0.0263	0.0096	0.0009	0.006	0.0015	0.0004	0.5751	0.0008	0.1315	0.0177	0.0116	0.0331	0.0011	0.0002	0.0006	0.4962	0.1315	0.5751	N	0.4436
82	M	0	0	0	0.0112	0	0	0	0	0	0	0.9549	0	0	0.0338	0	0	0	0	0	0	0.07	0.9549	0.9549	M	0
83	D	0	0	0.9999	0	0	0	0	0	0	0	0	0	0	0	0	0	0	0	0	0	0.0002	0.9999	0.9999	D	0
84	G	0	0	0	0	0	1	0	0	0	0	0	0	0	0	0	0	0	0	0	0	0.0002	1	1	G	0
85	V	0	0	0	0	0	0	0	0	0	0	0	0	0	0	0	0	0	0.9999	0	0	0.0003	0.9999	0.9999	V	0
86	P	0.0006	0	0	0	0	0	0	0	0	0.0007	0	0	0.9973	0.0013	0	0	0	0	0	0	0.0074	0.9973	0.9973	P	0
87	G	0	0.0008	0.0144	0	0	0.9848	0	0	0	0	0	0	0	0	0	0	0	0	0	0	0.0275	0.9848	0.9848	G	0
88	I	0.0002	0.0001	0	0	0.0461	0	0	0.2653	0	0.6359	0.0338	0	0	0	0	0.0039	0.0001	0.0142	0	0.0001	0.3289	0.2653	0.6359	L	0.3707
89	S	0	0	0	0	0	0	0	0	0	0	0	0	0	0	0	0.9999	0	0	0	0	0.0003	0.9999	0.9999	S	0
90	Y	0	0	0	0	0.9708	0	0	0.0001	0	0.0002	0	0	0	0	0	0	0	0.001	0.0001	0.0277	0.0468	0.0277	0.9708	F	0.943
91	K	0.1033	0	0.2918	0.1033	0.0001	0.0019	0.1845	0.0001	0.149	0.0001	0.0209	0.0309	0.0535	0.0129	0.0187	0.0216	0.0031	0.0009	0	0.0034	0.6812	0.149	0.2918	D	0.1427
92	G	0.0007	0.0006	0.0006	0	0	0.9942	0	0	0	0	0	0.0039	0	0	0	0	0	0	0	0	0.014	0.9942	0.9942	G	0
93	I	0	0	0	0	0	0	0	0.9944	0	0	0	0	0	0	0	0	0	0.0055	0	0	0.0117	0.9944	0.9944	I	0
94	A	0.5045	0	0.0041	0.2496	0	0.017	0.0068	0	0.0284	0.0001	0.0326	0.0015	0.1219	0.012	0.0174	0.0009	0.0019	0	0	0.0013	0.4833	0.5045	0.5045	A	0
95	P	0.1186	0	0	0.0001	0	0.0001	0.0001	0	0	0	0	0.0029	0.8519	0.002	0.0186	0.0042	0.0013	0	0	0	0.1768	0.8519	0.8519	P	0
96	G	0.0001	0	0.2788	0.0001	0	0.621	0.0088	0	0.0043	0.0001	0	0.0755	0	0.0001	0.0078	0.0032	0.0001	0	0	0.0001	0.3251	0.621	0.621	G	0
97	E	0.0007	0	0.0366	0.326	0	0.5093	0.0001	0	0	0	0	0.0007	0	0.0152	0.0001	0.1043	0.0069	0	0	0	0.3931	0.326	0.5093	G	0.1833
98	T	0.0061	0.0001	0.0283	0.0029	0.0007	0.0028	0.0001	0.0024	0.0001	0.1925	0.1854	0.0002	0.0001	0.0094	0.0068	0.0315	0.4511	0.0794	0	0.0001	0.5234	0.4511	0.4511	T	0
99	F	0	0	0	0	0.4283	0	0.0029	0.0001	0	0.0002	0	0	0	0	0	0	0	0.0001	0.0022	0.5661	0.2406	0.4283	0.5661	Y	0.1378
100	T	0.0064	0	0.0041	0.2167	0.0004	0	0.0128	0.0056	0.0088	0.0485	0	0.0021	0.0031	0.0051	0.0076	0.0001	0.2664	0.4123	0	0.0001	0.4931	0.2664	0.4123	V	0.1459
101	Y	0	0	0	0	0	0	0.0103	0	0	0	0	0	0	0	0	0	0	0	0	0.9897	0.0193	0.9897	0.9897	Y	0
102	R	0.0001	0.0006	0.0001	0.0022	0	0	0.03	0	0.1565	0.0001	0	0.0001	0	0.2077	0.5404	0.0144	0.0472	0	0.0005	0	0.4294	0.5404	0.5404	R	0
103	F	0	0	0	0	0.998	0	0	0.002	0	0	0	0	0	0	0	0	0	0	0	0	0.005	0.998	0.998	F	0
104	K	0.0107	0	0.0236	0.0056	0	0	0.0084	0.0006	0.6404	0.0081	0.0006	0.0041	0.1345	0.0395	0.0218	0.0001	0.1021	0	0	0.0001	0.4265	0.6404	0.6404	K	0
105	V	0.0382	0.0001	0	0	0	0	0	0.0328	0	0.2007	0	0	0	0	0	0	0.0002	0.7278	0	0	0.2655	0.7278	0.7278	V	0
106	Q	0.0039	0.0004	0.0003	0.0041	0.0001	0.0002	0.0347	0.023	0.2499	0.0088	0.002	0.0534	0.0069	0.1161	0.4432	0.0008	0.0059	0.0457	0.0003	0.0001	0.5471	0.1161	0.4432	R	0.3271
107	Q	0	0	0	0	0	0	0	0	0	0	0	0	0	0.9999	0	0	0	0	0	0	0.0003	0.9999	0.9999	Q	0
108	T	0.0441	0	0	0	0	0.0212	0.1435	0	0	0	0	0.37	0	0	0	0.3986	0.0211	0	0	0.0011	0.4422	0.0211	0.3986	S	0.3775
109	G	0	0	0	0	0	0.9988	0	0	0	0	0	0	0	0	0	0.0012	0	0	0	0	0.0032	0.9988	0.9988	G	0
110	T	0	0	0	0	0	0	0	0	0	0	0	0	0	0	0	0.0149	0.9851	0	0	0	0.026	0.9851	0.9851	T	0
111	Y	0	0	0	0	0	0	0.0012	0	0	0	0	0	0	0	0	0	0	0	0	0.9988	0.0033	0.9988	0.9988	Y	0
112	W	0	0	0	0	0	0	0	0	0	0	0	0	0	0	0	0	0	0	0.9999	0	0.0003	0.9999	0.9999	W	0
113	Y	0	0	0	0	0	0	0	0	0	0	0	0	0	0	0	0	0	0	0	0.9999	0.0003	0.9999	0.9999	Y	0

114	H	0	0	0	0	0	0	0.9988	0	0	0	0	0	0	0.0011	0	0	0	0	0	0	0.0032	0.9988	0.9988	H	0
115	S	0.0525	0	0	0	0	0.0093	0	0	0	0	0	0	0	0	0	0.9379	0.0001	0	0	0	0.0871	0.9379	0.9379	S	0
116	H	0	0	0	0	0	0	0.9999	0	0	0	0	0	0	0	0	0	0	0	0	0	0.0003	0.9999	0.9999	H	0
117	S	0	0	0	0	0	0	0	0	0	0	0	0	0	0	0.0014	0.9231	0.0754	0	0	0	0.093	0.9231	0.9231	S	0
118	G	0.0318	0.0003	0.0013	0.0011	0.0006	0.8514	0.0005	0.0007	0.0011	0.005	0.0026	0.0013	0.0008	0.0007	0.0753	0.0175	0.006	0.0012	0.0002	0.0005	0.2194	0.8514	0.8514	G	0
119	M	0.0028	0	0	0	0.6143	0.0013	0.0057	0.0001	0	0.3	0.0587	0	0	0	0	0	0.0011	0.0038	0.0001	0.012	0.3225	0.0587	0.6143	F	0.5557
120	Q	0	0	0	0.0035	0	0	0.0108	0	0	0	0	0	0	0.9847	0.0007	0	0	0	0	0	0.0307	0.9847	0.9847	Q	0
121	E	0	0	0	0.9987	0	0	0	0	0	0	0	0	0	0.0012	0	0	0	0	0	0	0.0034	0.9987	0.9987	E	0
122	Q	0.0085	0	0	0	0	0	0	0	0	0	0.0542	0	0	0.9373	0	0	0	0	0	0	0.0868	0.9373	0.9373	Q	0
123	T	0.2339	0.0001	0.0019	0.0365	0.0001	0.0034	0.0001	0.0113	0.0026	0.0564	0.0001	0.0084	0.0001	0.0515	0.0149	0.1811	0.3216	0.0759	0	0.0001	0.618	0.3216	0.3216	T	0
124	G	0.0082	0	0	0	0	0.9918	0	0	0	0	0	0	0	0	0	0	0	0	0	0	0.016	0.9918	0.9918	G	0
125	M	0.0002	0.0001	0	0	0	0.0008	0	0.0146	0	0.2468	0.1616	0	0	0	0	0	0.0002	0.5756	0	0	0.3442	0.1616	0.5756	V	0.4141
126	Y	0	0	0	0	0.0107	0	0	0.0121	0	0.0495	0.0054	0	0	0	0.0363	0.0049	0	0.0001	0.0001	0.8806	0.1807	0.8806	0.8806	Y	0
127	G	0.0023	0	0	0	0	0.9977	0	0	0	0	0	0	0	0	0	0	0	0	0	0	0.0056	0.9977	0.9977	G	0
128	T	0.5051	0	0	0	0	0.0144	0	0	0	0	0	0	0.4613	0	0	0.0145	0.0046	0	0	0	0.2841	0.0046	0.5051	A	0.5005
129	I	0	0	0	0	0	0	0	0.3507	0	0.6393	0.0099	0	0	0	0	0	0	0	0	0	0.2338	0.3507	0.6393	L	0.2886
130	V	0	0	0	0	0	0	0	0.2646	0	0	0	0	0	0	0	0	0	0.7353	0	0	0.1932	0.7353	0.7353	V	0
131	I	0.0001	0	0	0	0	0	0	0.9385	0	0.0121	0	0	0	0	0	0	0.0001	0.0492	0	0	0.0882	0.9385	0.9385	I	0
132	E	0.0047	0	0.6326	0.3457	0	0	0.0001	0	0.0097	0.0001	0	0.0048	0	0.0012	0.0001	0.0001	0.0001	0	0	0.0008	0.2576	0.3457	0.6326	D	0.2869
133	P	0.4262	0	0	0.0007	0	0.0002	0	0	0	0	0	0	0.5611	0	0	0.0117	0	0	0	0	0.2496	0.5611	0.5611	P	0
134	A	0.163	0.0001	0.0064	0.014	0.0001	0.0016	0.0007	0.0248	0.4101	0.0403	0.0026	0.0004	0.0032	0.005	0.3188	0.0049	0.0004	0.0035	0	0.0001	0.4907	0.163	0.4101	K	0.2471
135	G	0.0439	0.0001	0.0998	0.551	0.0003	0.1727	0.0164	0.0004	0.0139	0.0007	0.0002	0.0183	0.0006	0.0225	0.0116	0.0224	0.0197	0.0051	0.0001	0.0002	0.516	0.1727	0.551	E	0.3783
136	G	0.0484	0.0001	0.0006	0.015	0.0003	0.1234	0.0002	0.0004	0.0385	0.0007	0.0002	0.0004	0.6645	0.0052	0.0874	0.0073	0.0052	0.0018	0.0001	0.0002	0.4024	0.1234	0.6645	P	0.5411
137	E	0.0084	0.0001	0.12	0.81	0.0001	0.0003	0.0016	0.0002	0.0187	0.0004	0.0062	0.003	0.0228	0.0003	0.0003	0.0003	0.0003	0.0003	0.0001	0.0069	0.247	0.81	0.81	E	0
138	T	0.0176	0	0	0	0	0	0	0.0231	0.0031	0.0001	0	0.0103	0.6593	0.0018	0.239	0.0133	0.0313	0.0009	0	0	0.3426	0.0313	0.6593	P	0.628
139	I	0.0024	0	0	0.0142	0.565	0	0.1406	0.0799	0	0.0009	0	0.0036	0	0.0185	0	0	0	0.0589	0.0032	0.1125	0.4702	0.0799	0.565	F	0.4851
140	R	0.107	0.0002	0.0068	0.0621	0.0004	0.0089	0.0266	0.0028	0.0608	0.0017	0.0012	0.0018	0.1658	0.1416	0.0854	0.2061	0.0998	0.0206	0.0001	0.0004	0.7444	0.0854	0.2061	S	0.1207
141	A	0.1721	0.0218	0.0006	0.0007	0.0433	0.0008	0.0024	0.0576	0.0006	0.0012	0.0003	0.0006	0.0018	0.0005	0.0007	0.0262	0.0313	0.0469	0.0088	0.5816	0.4877	0.1721	0.5816	Y	0.4095
142	D	0.0357	0	0.6975	0.1708	0	0	0.0042	0.0034	0.0001	0.0001	0.0024	0.0071	0	0.0776	0.0001	0.0008	0.0001	0	0	0.0001	0.3247	0.6975	0.6975	D	0
143	R	0.0001	0.0046	0	0.0806	0	0	0.0001	0	0.0341	0.0001	0	0.0165	0	0.0159	0.8243	0.0071	0.0164	0	0	0	0.2479	0.8243	0.8243	R	0
144	D	0	0	0.74	0.26	0	0	0	0	0	0	0	0	0	0	0	0	0	0	0	0	0.1915	0.74	0.74	D	0
145	Y	0	0.0045	0	0	0.0179	0	0.3728	0.0001	0	0.0103	0	0	0	0.0194	0	0	0	0.0001	0.0001	0.5747	0.3039	0.5747	0.5747	Y	0
146	V	0.0002	0.0001	0	0	0	0	0	0.0182	0	0.0055	0	0	0	0	0	0	0.0245	0.9513	0	0	0.0818	0.9513	0.9513	V	0
147	V	0.0002	0.0001	0	0	0	0	0	0.1999	0	0.0558	0.0058	0	0	0	0	0	0.0002	0.7378	0	0	0.2483	0.7378	0.7378	V	0
148	Q	0.0002	0.0001	0	0	0.0326	0	0	0.0055	0	0.3121	0.5012	0	0	0.0528	0	0	0.0061	0.089	0	0.0001	0.4198	0.0528	0.5012	M	0.4484
149	L	0	0	0	0	0.0242	0	0	0.0236	0	0.9506	0.0001	0	0	0	0	0	0	0.001	0.0001	0.0003	0.08	0.9506	0.9506	L	0
150	S	0	0	0	0	0	0.0047	0	0	0	0	0	0.0467	0	0	0	0.7788	0.1697	0	0	0	0.2221	0.7788	0.7788	S	0
151	D	0	0	0.9988	0	0	0.0012	0	0	0	0	0	0	0	0	0	0	0	0	0	0	0.0032	0.9988	0.9988	D	0
152	W	0	0	0	0	0	0	0	0	0	0	0	0	0	0	0	0	0	0	0.9999	0	0.0003	0.9999	0.9999	W	0
153	T	0.0067	0	0	0	0	0.0002	0	0	0	0	0.0185	0	0	0	0	0.2131	0.7613	0	0	0	0.2165	0.7613	0.7613	T	0
154	D	0.0001	0	0.9078	0.0007	0.0088	0.0008	0.0517	0	0.0001	0.0001	0	0.0005	0	0.0001	0.0001	0.0249	0.0001	0	0	0.0042	0.1396	0.9078	0.9078	D	0
155	E	0.0153	0	0.046	0.75	0	0.0205	0.0001	0	0.0045	0.0309	0.0229	0.0001	0	0.0306	0.0765	0.0014	0.0011	0	0	0.0001	0.3481	0.75	0.75	E	0
156	D	0.0333	0	0.5725	0.0447	0.0001	0.0042	0.0029	0.0001	0.1021	0.0001	0	0.1572	0.0002	0.0086	0.0069	0.0421	0.0047	0.0204	0	0.0001	0.4852	0.5725	0.5725	D	0
157	P	0.0275	0	0	0	0	0.0023	0	0	0	0.0007	0	0	0.966	0	0	0.0033	0	0	0	0	0.0574	0.966	0.966	P	0
158	M	0.2289	0.0003	0.0136	0.1469	0.0002	0.0137	0.1155	0.0084	0.0042	0.0286	0.1223	0.0109	0.0001	0.0885	0.0062	0.0325	0.0593	0.1152	0.0049	0.0001	0.7551	0.1223	0.2289	A	0.1066
159	R	0.1186	0.0001	0.0641	0.0254	0.0001	0.0241	0.0784	0.0002	0.0211	0.0004	0.0001	0.0776	0.0002	0.059	0.3607	0.1035	0.0515	0.006	0.0023	0.0066	0.7011	0.3607	0.3607	R	0
160	V	0.0037	0.0001	0	0	0.0001	0	0	0.2256	0	0.4635	0.0011	0	0	0	0	0	0.0002	0.3056	0	0	0.3632	0.3056	0.4635	L	0.1578
161	F	0.0019	0.0026	0	0	0.157	0	0	0.0284	0	0.3778	0.298	0	0	0.0033	0	0	0.0001	0.0138	0	0.1168	0.4942	0.157	0.3778	L	0.2208
162	S	0.3461	0	0.0118	0.0002	0	0.0181	0.0275	0	0.3341	0.0103	0	0.0087	0.0023	0.0094	0.1033	0.1117	0.0141	0.0001	0	0.0021	0.5543	0.1117	0.3461	A	0.2344
163	K	0.0007	0	0	0.0001	0	0	0.0035	0.0094	0.5453	0.0767	0.0039	0.0502	0	0.0019	0.0721	0.0022	0.2334	0.0005	0	0	0.4436	0.5453	0.5453	K	0
164	L	0	0	0	0	0	0	0	0	0	0.9899	0.0087	0	0	0	0	0	0	0.0013	0	0	0.0202	0.9899	0.9899	L	0
165	K	0	0	0	0	0	0	0	0	0.9696	0	0.0008	0	0	0	0.0294	0	0	0	0	0	0.0473	0.9696	0.9696	K	0
166	T	0.0044	0	0.0163	0.005	0.0068	0	0.0001	0.0432	0.7021	0.0108	0.003	0.0001	0	0.1051	0.0188	0.0112	0.029	0.044	0	0	0.4026	0.029	0.7021	K	0.6732
167	Q	0.0001	0	0.0299	0.0113	0	0	0.0077	0	0.0054	0.0309	0.1045	0.0022	0	0.6538	0.1291	0.0188	0.0001	0.006	0	0.0001	0.4102	0.6538	0.6538	Q	0
168	S	0.1209	0	0	0	0	0.0628	0	0	0	0	0	0.0058	0.0346	0	0	0.7756	0.0001	0	0	0	0.2588	0.7756	0.7756	S	0
169	D	0.0001	0	0.8188	0.0064	0	0.0884	0.0415	0	0.0001	0.0001	0.0013	0.0124	0	0.0001	0.0001	0.0285	0.0022	0	0	0.0001	0.2423	0.8188	0.8188	D	0
170	Y	0.017	0	0	0	0.1221	0	0.0092	0.0209	0	0.0002	0.0029	0	0	0	0	0	0.0036	0.0349	0.0001	0.7889	0.2655	0.7889	0.7889	Y	0
171	Y	0	0	0.1603	0	0.0608	0	0	0	0	0	0	0	0	0	0	0	0	0	0	0.7788	0.2201	0.7788	0.7788	Y	0
172	N	0	0	0	0	0	0	0	0	0	0	0	0.9999	0	0	0	0	0	0	0	0	0.0003	0.9999	0.9999	N	0
173	F	0.0007	0	0.0028	0.0026	0.4157	0.0023	0.0107	0	0.0001	0.0504	0.007	0.0766	0	0.0701	0.0398	0.0012	0.0145	0.0014	0	0.3041	0.5353	0.4157	0.4157	F	0
174	N	0.0647	0	0.0071	0.0001	0	0.0752	0.4161	0.014	0.0065	0.0001	0	0.3085	0	0.044	0.0127	0.0199	0.0001	0	0	0.0308	0.5372	0.3085	0.4161	H	0.1076
175	Q	0.005	0	0	0.0747	0	0	0	0	0.5264	0.0767	0.0189	0	0.0024	0.2546	0.0411	0	0	0.0001	0	0	0.443	0.2546	0.5264	K	0.2718
176	P	0	0	0	0.0001	0	0	0.0008	0	0.0001	0.0146	0	0.0001	0.3974	0.0132	0.5735	0	0	0	0	0	0.272	0.3974	0.5735	R	0.1761
177	T	0.0037	0	0.0792	0.0002	0.0001	0.0001	0.0002	0.0007	0.0001	0.0002	0.0001	0.0008	0.0001	0.0002	0.0002	0.0031	0.9107	0.0001	0	0.0001	0.1173	0.9107	0.9107	T	0
178	V	0.0997	0.0001	0.0001	0.0002	0.0937	0.0001	0.0149	0.0013	0.0001	0.1215	0.0029	0.0001	0.0001	0.0001	0.0001	0.0002	0.0002	0.6645	0	0.0001	0.3611	0.6645	0.6645	V	0
179	A	0.0533	0.0001	0.006	0.0039	0.0317	0.6468	0.0003	0.0058	0.089	0.0042	0.0001	0.001	0.0651	0.0044	0.0098	0.0275	0.0147	0.0361	0.0001	0.0002	0.4703	0.0533	0.6468	G	0.5935
180	D	0.007	0	0.7792	0.0362	0.0001	0.0107	0.0002	0.0001	0.0799	0.0001	0	0.0086	0.0002	0.0111	0.0109	0.0262	0.0292	0.0001	0	0.0001	0.316	0.7792	0.7792	D	0
181	F	0	0	0	0	0.856	0	0	0.0001	0	0.121	0	0	0	0	0	0	0.0188	0.0035	0.0001	0.0003	0.1634	0.856	0.856	F	0
182	F	0.0298	0.0001	0	0	0.2036	0	0	0.4457	0.0204	0.1425	0.0463	0	0	0	0	0.0049	0.0097	0.092	0.0048	0.0001	0.5368	0.2036	0.4457	I	0.2421
183	R	0.0526	0	0.2628	0.0303	0	0.0011	0.0663	0.0001	0.0256	0.0001	0	0.1693	0.0001	0.0107	0.2927	0.0839	0.0028	0.0015	0	0.0001	0.6144	0.2927	0.2927	R	0
184	D	0.0039	0	0.9615	0.0346	0	0	0	0	0	0	0	0	0	0	0	0	0	0	0	0	0.0588	0.9615	0.9615	D	0
185	V	0.1108	0.0001	0	0	0.0144	0	0	0.1499	0	0.0009	0.0042	0	0	0	0	0.0057	0.0043	0.7095	0	0	0.3062	0.7095	0.7095	V	0
186	S	0.183	0.0015	0.0058	0.0194	0	0.1482	0.0063	0	0.073	0.0001	0	0.026	0	0.0153	0.1536	0.354	0.0136	0	0	0.0001	0.6038	0.354	0.354	S	0
187	R	0.153	0	0.1095	0.3475	0.0001	0.0007	0.0031	0.0002	0.1181	0.0126	0.0001	0.024	0.0004	0.0405	0.1345	0.0217	0.0336	0.0003	0	0.0001	0.6424	0.1345	0.3475	E	0.213
188	N	0.0006	0.0001	0.0789	0.0455	0.0001	0.0016	0.0288	0.0102	0.3491	0.0059	0.0943	0.1461	0.0002	0.0898	0.0269	0.0232	0.0809	0.0126	0	0.005	0.6997	0.1461	0.3491	K	0.203
189	G	0	0	0.0793	0	0	0.9094	0	0	0	0	0	0.0013	0	0	0	0.0099	0	0	0	0	0.1143	0.9094	0.9094	G	0
190	F	0.0026	0.0001	0	0	0.0677	0.0021	0	0.024	0	0.3077	0.0224	0	0	0	0	0	0.0065	0.0496	0.5169	0.0001	0.4257	0.0677	0.5169	W	0.4492
191	K	0.1807	0.0001	0.0145	0.053	0.0061	0.1549	0.0018	0.0002	0.0942	0.0072	0.0041	0.0045	0.0002	0.0408	0.0823	0.3359	0.0192	0.0003	0	0.0002	0.6504	0.0942	0.3359	S	0.2417
192	A	0.5035	0	0.0246	0.0941	0	0.0339	0.0043	0	0.0948	0.0001	0	0.0574	0.0117	0.0441	0.0035	0.0761	0.0517	0	0	0.0001	0.5828	0.5035	0.5035	A	0
193	A	0.4333	0	0	0	0	0.0003	0	0	0	0	0	0.0007	0	0	0	0.0056	0.5599	0	0	0	0.2424	0.4333	0.5599	T	0.1266
194	L	0.0353	0.0001	0	0	0.0938	0	0.0033	0.0945	0	0.3457	0.0482	0	0	0	0	0	0.0728	0.2499	0.036	0.0202	0.6123	0.3457	0.3457	L	0
195	D	0.4997	0	0.1449	0.0434	0	0.0151	0.0001	0	0.0342	0.0001	0.0005	0.0314	0	0.0376	0.0512	0.117	0.0242	0.0004	0	0.0001	0.5599	0.1449	0.4997	A	0.3548
196	N	0.0032	0	0.5729	0.096	0.0001	0.0004	0.0002	0.0027	0.1844	0.0052	0.039	0.0527	0.0002	0.0028	0.0188	0.0152	0.0057	0.0005	0	0.0001	0.4657	0.0527	0.5729	D	0.5202
197	R	0	0	0	0	0	0	0.0017	0	0	0	0	0	0	0	0.9846	0.0016	0	0	0.012	0	0.0301	0.9846	0.9846	R	0
198	K	0.0434	0	0.0001	0.0101	0	0.02	0.0109	0	0.5302	0.0705	0.0032	0.0001	0	0.0483	0.069	0.0137	0.0716	0.0009	0.1079	0.0001	0.5609	0.5302	0.5302	K	0
199	M	0.0666	0	0.0011	0.0118	0	0.0032	0.0001	0.0065	0.0001	0.0019	0.8884	0.0008	0	0.0026	0.0001	0.0091	0.0056	0.0021	0	0	0.173	0.8884	0.8884	M	0
200	W	0	0.0018	0	0	0	0	0	0	0	0.0014	0	0	0	0	0	0	0	0	0.9968	0	0.008	0.9968	0.9968	W	0
201	N	0.536	0	0	0.0251	0	0.0771	0	0	0	0	0	0.332	0	0.0242	0	0.0023	0.0026	0.0004	0	0	0.3723	0.332	0.536	A	0.2039
202	Q	0.0284	0	0.013	0.3408	0	0.0116	0.0002	0.0001	0.1147	0.005	0.0052	0.0002	0.0001	0.3473	0.1113	0.0074	0.0116	0.0031	0	0.0001	0.5349	0.3473	0.3473	Q	0
203	M	0	0	0	0	0	0.0103	0	0	0	0	0.9867	0	0	0	0	0	0.0018	0.0011	0	0	0.0267	0.9867	0.9867	M	0
204	R	0.0001	0	0	0.0001	0	0.0306	0.0028	0	0.3536	0.0001	0	0.0595	0	0.0201	0.5296	0.0001	0.0032	0	0	0	0.3662	0.5296	0.5296	R	0
205	M	0	0	0	0	0	0	0	0.0011	0	0	0.9988	0	0	0	0	0	0	0	0	0	0.0032	0.9988	0.9988	M	0
206	S	0.0029	0.0001	0.0773	0.0186	0.0001	0.0105	0.0002	0.0001	0.0002	0.0001	0.0832	0.3589	0	0.0001	0.0001	0.3599	0.0672	0.0204	0	0.0001	0.5173	0.3599	0.3599	S	0
207	P	0.0028	0	0.0383	0.0001	0	0	0.0001	0	0.0001	0.0005	0.0111	0.0001	0.8979	0.0284	0.0103	0.01	0.0001	0	0	0.0001	0.1643	0.8979	0.8979	P	0
208	T	0.0154	0	0	0	0	0.0049	0	0	0	0.0049	0	0.0164	0	0	0.0623	0.0241	0.8718	0	0	0	0.1899	0.8718	0.8718	T	0
209	D	0	0	0.9988	0	0	0	0	0	0	0	0	0.0012	0	0	0	0	0	0	0	0	0.0033	0.9988	0.9988	D	0
210	L	0.0001	0	0	0	0.1289	0	0	0.103	0	0.763	0.0027	0	0	0.0005	0	0	0	0.0002	0.0001	0.0012	0.2467	0.763	0.763	L	0
211	A	0.6863	0	0	0	0	0.0343	0	0	0	0.0068	0.0161	0.008	0	0	0	0.1746	0.0702	0.0034	0	0	0.3425	0.6863	0.6863	A	0
212	D	0	0	0.9987	0	0	0.0012	0	0	0	0	0	0	0	0	0	0	0	0	0	0	0.0034	0.9987	0.9987	D	0
213	V	0.001	0.0001	0	0	0	0	0	0.0777	0	0.1841	0	0	0	0	0	0	0.0002	0.7367	0	0	0.2493	0.7367	0.7367	V	0
214	S	0.0042	0	0.0021	0	0	0.0189	0	0	0	0	0	0.0461	0	0	0	0.7883	0.1402	0	0	0	0.2396	0.7883	0.7883	S	0
215	G	0.1535	0	0	0	0	0.8314	0	0	0	0	0	0.0041	0	0	0	0.0052	0.0055	0	0	0	0.1743	0.8314	0.8314	G	0
216	Y	0.4072	0.0002	0.0075	0.0381	0.0054	0.0081	0.0405	0.0004	0.0458	0.0071	0.0002	0.0697	0.0003	0.0206	0.0005	0.0242	0.0249	0.0153	0.0003	0.2839	0.5954	0.2839	0.4072	A	0.1233
217	T	0.0424	0.0001	0.0003	0.0003	0.0001	0.0088	0.0002	0.0002	0.0003	0.0003	0.0006	0.0016	0.0002	0.0002	0.0003	0.0156	0.9222	0.006	0	0.0001	0.1282	0.9222	0.9222	T	0
218	Y	0.0169	0	0	0	0.0799	0	0	0.0001	0	0.0447	0.0297	0	0	0.0036	0	0	0	0.0001	0.0001	0.8247	0.2329	0.8247	0.8247	Y	0
219	T	0.0007	0	0	0	0	0	0	0	0	0	0	0	0	0	0.0227	0.0042	0.9723	0	0	0	0.0475	0.9723	0.9723	T	0
220	Y	0	0	0	0	0.1223	0	0.0009	0.0001	0	0.0001	0	0.0034	0	0	0	0	0	0.0001	0.0001	0.8728	0.1357	0.8728	0.8728	Y	0
221	L	0	0	0	0	0	0	0	0	0	0.9983	0	0	0	0.0009	0.0008	0	0	0	0	0	0.0048	0.9983	0.9983	L	0
222	V	0.0267	0.0001	0	0	0	0	0	0.0996	0	0.2176	0.5749	0	0	0	0	0	0.016	0.0649	0	0	0.4083	0.0649	0.5749	M	0.5099
223	N	0	0	0	0	0	0	0	0	0	0	0	0.9999	0	0	0	0	0	0	0	0	0.0003	0.9999	0.9999	N	0
224	G	0	0	0	0	0	1	0	0	0	0	0	0	0	0	0	0	0	0	0	0	0.0002	1	1	G	0
225	T	0.0546	0.0099	0.0001	0.0023	0.0088	0.0001	0.0572	0	0.107	0.0137	0.0041	0.0124	0	0.5018	0.0591	0.0129	0.0921	0.0625	0	0.0014	0.5918	0.0921	0.5018	Q	0.4097
226	T	0.461	0	0	0	0	0.0126	0	0	0	0	0	0.0266	0.1594	0	0.0042	0.0803	0.2534	0.0023	0	0	0.4641	0.2534	0.461	A	0.2075
227	P	0.0404	0	0	0	0	0.0002	0	0	0	0	0	0	0.8542	0	0	0.0238	0.0813	0	0	0	0.1872	0.8542	0.8542	P	0
228	A	0.3101	0	0.3883	0.0245	0.0001	0.0556	0.0011	0.0001	0.007	0.0675	0.0106	0.1014	0.0002	0.0025	0.004	0.0095	0.0174	0.0001	0	0.0001	0.5484	0.3101	0.3883	D	0.0783
229	G	0.1472	0.0001	0.035	0.0065	0.0002	0.4192	0.0231	0.0001	0.0506	0.006	0.1386	0.0185	0.0003	0.0293	0.0239	0.0615	0.0386	0.0009	0	0.0006	0.6408	0.4192	0.4192	G	0

230	N	0.0043	0	0.0085	0.0001	0	0.0279	0.0001	0	0.0008	0.0001	0	0.9428	0.0057	0.0095	0.0001	0.0001	0.0001	0	0	0	0.1017	0.9428	0.9428	N	0
231	W	0	0	0	0	0.049	0	0	0	0	0.0001	0	0	0	0	0	0	0	0	0.9444	0.0062	0.0792	0.9444	0.9444	W	0
232	T	0.0293	0	0.0001	0.0058	0	0.0001	0.0001	0.004	0.0192	0.0001	0	0.0042	0.0001	0.0001	0.0208	0.0369	0.8555	0.0194	0	0.0042	0.2325	0.8555	0.8555	T	0
233	G	0.0677	0.0297	0	0	0	0.8027	0	0	0.007	0	0	0	0	0.0736	0	0.0007	0.01	0.0005	0.0079	0	0.262	0.8027	0.8027	G	0
234	L	0.0105	0.0001	0.0043	0	0.0004	0	0	0.1318	0.0042	0.6762	0.0183	0	0.0163	0.0084	0	0.0024	0.0067	0.1202	0	0.0001	0.3722	0.6762	0.6762	L	0
235	F	0.0184	0.0012	0	0	0.8824	0	0	0.0207	0	0.0002	0	0	0	0	0	0	0.0001	0.0766	0.0001	0.0002	0.1587	0.8824	0.8824	F	0
236	R	0.0151	0.0001	0.0051	0.0316	0.0001	0.0007	0.0013	0.0007	0.443	0.0007	0.0001	0.0351	0.0002	0.0821	0.3299	0.0205	0.0136	0.0197	0	0.0002	0.4991	0.3299	0.443	K	0.1131
237	P	0.1596	0	0.0001	0.0001	0	0	0.0073	0	0.0718	0.001	0	0.0001	0.7023	0.0006	0.017	0.025	0.0078	0.0072	0	0	0.3392	0.7023	0.7023	P	0
238	G	0	0	0.0009	0	0	0.9668	0	0	0	0	0	0	0	0	0.0289	0.0018	0	0	0.0016	0	0.0545	0.9668	0.9668	G	0
239	E	0	0	0.0333	0.849	0	0.0027	0	0	0.0163	0	0	0	0	0.0984	0	0	0	0	0	0	0.1889	0.849	0.849	E	0
240	K	0.0042	0.0006	0	0	0	0	0.0041	0	0.5396	0.0001	0.0008	0	0.0807	0.0024	0.298	0	0.0692	0	0	0	0.3859	0.5396	0.5396	K	0
241	V	0.0165	0.0001	0	0	0.0002	0	0	0.3335	0	0.2467	0.0025	0	0	0	0	0.0001	0.0002	0.4	0	0.0001	0.39	0.4	0.4	V	0
242	R	0	0	0	0	0	0	0	0	0.0949	0.013	0	0	0	0.0001	0.8917	0	0	0	0	0	0.1289	0.8917	0.8917	R	0
243	L	0	0	0	0	0	0	0	0	0	0.9874	0.001	0	0	0	0	0	0	0.0115	0	0	0.0239	0.9874	0.9874	L	0
244	R	0	0	0	0	0	0	0	0	0	0	0	0	0	0.0012	0.9975	0.0012	0	0	0	0	0.0065	0.9975	0.9975	R	0
245	F	0	0	0	0	0.7453	0	0	0.0141	0	0.2255	0.0007	0	0	0	0	0	0	0.0138	0.0001	0.0003	0.2286	0.7453	0.7453	F	0
246	V	0	0	0	0	0	0	0	0.9043	0	0	0	0	0	0	0	0	0	0.0957	0	0	0.1056	0.0957	0.9043	I	0.8086
247	N	0	0.0103	0.0009	0	0	0	0	0	0	0	0	0.9795	0	0	0	0.0093	0	0	0	0	0.0392	0.9795	0.9795	N	0
248	S	0.2356	0	0	0	0	0.7349	0	0	0	0	0	0	0	0	0	0.0292	0.0001	0	0	0	0.2248	0.0292	0.7349	G	0.7057
249	G	0.1097	0.0018	0	0	0	0.0244	0	0	0	0	0	0	0	0	0	0.8639	0.0001	0	0	0	0.1581	0.0244	0.8639	S	0.8395
250	A	0.7292	0	0	0	0	0.0367	0	0	0	0	0	0	0	0	0	0.2163	0.0176	0	0	0	0.2525	0.7292	0.7292	A	0
251	M	0.0118	0	0.0001	0.0001	0	0	0.0001	0	0.0001	0.0001	0.7314	0.1101	0	0.0984	0.0001	0.0191	0.0285	0	0	0	0.3124	0.7314	0.7314	M	0
252	T	0	0	0	0	0	0	0	0	0	0	0	0	0	0	0	0.3041	0.6958	0	0	0	0.2053	0.6958	0.6958	T	0
253	F	0	0	0	0	0.1876	0	0.0173	0.1836	0	0.0192	0	0	0	0	0	0	0.012	0.0001	0.0001	0.5799	0.3819	0.1876	0.5799	Y	0.3923
254	F	0	0	0	0	0.9396	0	0	0.0001	0	0.0059	0	0	0	0	0	0	0	0.0001	0.0001	0.054	0.0838	0.9396	0.9396	F	0
255	D	0	0	0.9999	0	0	0	0	0	0	0	0	0	0	0	0	0	0	0	0	0	0.0002	0.9999	0.9999	D	0
256	V	0.0002	0.0001	0	0	0.0526	0	0	0.0628	0	0.0555	0.0013	0	0	0	0	0	0.0001	0.8271	0	0.0001	0.2207	0.8271	0.8271	V	0
257	R	0	0	0	0	0	0	0.0016	0	0	0	0	0	0	0	0.9969	0.0015	0	0	0	0	0.0079	0.9969	0.9969	R	0
258	I	0	0	0	0	0	0	0	0.9792	0	0.0023	0	0	0	0	0	0	0	0.0185	0	0	0.0365	0.9792	0.9792	I	0
259	P	0	0	0	0	0	0	0	0	0	0.0028	0	0	0.9971	0	0	0	0	0	0	0	0.0067	0.9971	0.9971	P	0
260	G	0	0	0.0005	0.0243	0	0.9677	0	0	0	0	0	0.0048	0	0	0	0.0026	0	0	0	0	0.0561	0.9677	0.9677	G	0
261	L	0	0	0	0	0	0	0	0.0029	0	0.9847	0	0	0	0	0	0	0	0.0123	0	0	0.0292	0.9847	0.9847	L	0
262	K	0.0009	0	0.0141	0.0039	0	0.0023	0.0001	0	0.9273	0.0001	0	0.0001	0.0126	0.0024	0.03	0	0.006	0	0	0	0.1275	0.9273	0.9273	K	0
263	M	0	0	0	0	0.0033	0	0	0.0123	0	0.186	0.7978	0	0	0	0	0	0	0.0001	0.0001	0.0002	0.1907	0.7978	0.7978	M	0
264	T	0.0006	0	0	0	0	0.0035	0	0.0091	0.057	0.0001	0.0019	0.0138	0	0.0119	0.0221	0.0288	0.849	0.0021	0	0	0.2319	0.849	0.849	T	0
265	V	0.0002	0.0001	0	0	0	0	0	0.0104	0	0.0093	0.0164	0	0	0	0	0	0.0002	0.9632	0	0	0.067	0.9632	0.9632	V	0
266	V	0.0002	0.0001	0	0	0	0	0	0.1802	0	0.0013	0	0	0	0	0	0	0.0001	0.818	0	0	0.1625	0.818	0.818	V	0
267	Q	0.8306	0	0	0	0	0.0258	0.005	0	0	0	0	0	0	0.0756	0	0.0628	0.0001	0	0	0	0.2159	0.0756	0.8306	A	0.7551
268	A	0.697	0.0001	0	0	0	0.0002	0	0.0001	0	0.0001	0.0141	0	0	0	0	0.1337	0.108	0.0467	0	0	0.3237	0.697	0.697	A	0
269	D	0	0	0.9999	0	0	0	0	0	0	0	0	0	0	0	0	0	0	0	0	0	0.0002	0.9999	0.9999	D	0
270	G	0	0	0	0	0	1	0	0	0	0	0	0	0	0	0	0	0	0	0	0	0.0002	1	1	G	0
271	Q	0.0001	0	0	0.0001	0	0.012	0.0001	0.0549	0	0.1784	0.0008	0.0517	0	0.6964	0.0001	0.0001	0.0001	0.0051	0	0	0.3221	0.6964	0.6964	Q	0
272	D	0.0031	0	0.2666	0.009	0.001	0.001	0.2948	0	0.0001	0.0101	0	0.097	0.1193	0.0013	0.0107	0.0032	0.0027	0	0	0.1801	0.5723	0.2666	0.2948	H	0.0282
273	V	0.0002	0.0001	0	0	0	0	0	0.101	0	0.0074	0	0	0	0	0	0	0.0001	0.891	0	0	0.1257	0.891	0.891	V	0
274	E	0.0752	0	0.0351	0.3242	0.0001	0.0007	0.0726	0.0002	0.2794	0.0032	0.0001	0.0516	0.0003	0.076	0.0145	0.0168	0.0295	0.0204	0	0.0001	0.6401	0.3242	0.3242	E	0
275	P	0.0008	0	0	0	0	0	0.0103	0	0	0	0	0	0.988	0	0	0.0009	0	0	0	0	0.0239	0.988	0.988	P	0
276	V	0.0012	0.0001	0	0	0	0	0	0.0138	0	0.0047	0.0007	0	0	0	0	0	0.0002	0.9791	0	0	0.0409	0.9791	0.9791	V	0
277	T	0.0748	0.0001	0.0605	0.0505	0.0001	0.0122	0.0001	0.0018	0.0051	0.0082	0.0001	0.0019	0.0384	0.0169	0.0076	0.4467	0.2451	0.0298	0	0.0001	0.5684	0.2451	0.4467	S	0.2017
278	V	0.0012	0.0001	0	0	0.0141	0	0	0.1086	0	0.0002	0	0	0	0	0	0	0.0002	0.8755	0	0	0.1442	0.8755	0.8755	V	0
279	D	0	0	0.9562	0.0297	0	0	0.0141	0	0	0	0	0	0	0	0	0	0	0	0	0	0.0694	0.9562	0.9562	D	0
280	E	0	0	0.0854	0.9145	0	0	0	0	0	0	0	0	0	0	0	0	0	0	0	0	0.0976	0.9145	0.9145	E	0
281	I	0.0002	0.0064	0	0	0.6101	0	0	0.0382	0	0.3142	0.0072	0	0	0	0	0	0.0001	0.0233	0.0001	0.0001	0.3177	0.0382	0.6101	F	0.5719
282	R	0	0	0	0	0	0	0.0011	0	0	0	0	0	0	0	0.9988	0	0	0	0	0	0.0032	0.9988	0.9988	R	0
283	I	0.0001	0	0	0	0.0968	0	0	0.7896	0	0.0938	0.0057	0	0	0	0	0	0	0.0135	0.0001	0.0003	0.243	0.7896	0.7896	I	0
284	G	0.6173	0	0	0	0	0.291	0	0	0	0	0	0	0	0	0	0.088	0.0035	0	0	0	0.2982	0.291	0.6173	A	0.3264
285	V	0.0402	0.0001	0	0.0007	0	0.0017	0	0.0021	0	0.0002	0	0	0.1194	0	0	0.0031	0.0307	0.8018	0	0	0.2393	0.8018	0.8018	V	0
286	A	0.901	0	0	0	0	0.0979	0	0	0	0	0	0	0	0	0	0.0001	0	0.0009	0	0	0.1102	0.901	0.901	A	0
287	E	0	0	0	0.9999	0	0	0	0	0	0	0	0	0	0	0	0	0	0	0	0	0.0003	0.9999	0.9999	E	0
288	T	0	0	0	0	0	0	0	0.0103	0	0	0	0	0	0	0	0	0.9757	0.0138	0	0	0.044	0.9757	0.9757	T	0
289	Y	0	0.0229	0	0	0.2243	0	0	0.0007	0	0.0409	0	0	0	0	0	0	0	0.0083	0.0001	0.7027	0.2832	0.7027	0.7027	Y	0
290	D	0	0	0.9999	0	0	0	0	0	0	0	0	0	0	0	0	0	0	0	0	0	0.0002	0.9999	0.9999	D	0
291	V	0.0001	0	0	0	0	0	0	0.0041	0	0.0043	0	0	0	0	0	0	0.0001	0.9913	0	0	0.0194	0.9913	0.9913	V	0
292	I	0.0002	0.0001	0	0	0.0011	0	0	0.7719	0	0.1943	0.0008	0	0	0	0	0	0.0001	0.0312	0	0.0001	0.2157	0.7719	0.7719	I	0
293	V	0.0051	0.0001	0	0	0	0	0	0.0338	0	0.0001	0	0	0	0	0	0	0.0001	0.9606	0	0	0.0618	0.9606	0.9606	V	0
294	T	0.0109	0	0.0001	0.7357	0.0001	0.0001	0.0002	0.0103	0.0001	0.0016	0.0081	0.0036	0.0001	0.0494	0.0266	0.0272	0.1203	0.0055	0	0.0001	0.3423	0.1203	0.7357	E	0.6154
295	P	0.0021	0.0001	0	0.001	0	0.0026	0	0.0002	0	0.0029	0.0009	0	0.9698	0	0	0.0014	0.0001	0.0188	0	0	0.0589	0.9698	0.9698	P	0
296	K	0.1432	0.0006	0.0604	0.1348	0.0012	0.1135	0.0036	0.0065	0.0612	0.0045	0.0007	0.0139	0.0077	0.0686	0.0251	0.0917	0.2306	0.0307	0.0004	0.001	0.7606	0.0612	0.2306	T	0.1694
297	D	0.0937	0	0.3613	0.2784	0.0001	0.0302	0.0037	0.0002	0.0565	0.0032	0.0006	0.0286	0.0042	0.1125	0.0067	0.0123	0.0076	0.0003	0	0.0001	0.5868	0.3613	0.3613	D	0
298	G	0.0641	0	0.2317	0.1883	0.0001	0.142	0.0077	0.0002	0.0413	0.0004	0.0016	0.0167	0.026	0.0824	0.1732	0.0208	0.0025	0.0009	0.0001	0.0001	0.6902	0.142	0.2317	D	0.0896
299	A	0.9642	0	0	0	0	0.0003	0	0	0	0	0	0	0.0191	0	0	0.0012	0.008	0.007	0	0	0.0657	0.9642	0.9642	A	0
300	Y	0	0.0086	0	0	0.052	0	0.1019	0.0001	0	0.0002	0	0	0	0	0	0.001	0	0.0001	0.0012	0.8349	0.1994	0.8349	0.8349	Y	0
301	T	0.0235	0.0001	0	0	0	0	0	0.0027	0.001	0.0002	0	0	0	0	0	0.0055	0.9591	0.0078	0	0	0.0739	0.9591	0.9591	T	0
302	I	0.0002	0.0001	0	0	0.0042	0	0	0.43	0	0.4759	0.0003	0	0	0	0	0	0.0001	0.0888	0	0.0001	0.3218	0.43	0.4759	L	0.046
303	F	0	0	0	0	0.9849	0	0	0.0048	0	0.0011	0	0	0	0	0	0	0	0.0022	0.0001	0.0066	0.033	0.9849	0.9849	F	0
304	A	0.984	0.008	0	0	0	0.0001	0	0	0	0	0	0	0	0	0	0.0078	0	0	0	0	0.0316	0.984	0.984	A	0
305	Q	0.0015	0	0	0.0136	0	0	0	0	0	0.0008	0	0	0	0.9839	0	0	0	0	0	0	0.0305	0.9839	0.9839	Q	0
306	S	0.1449	0	0.0533	0	0	0.0071	0	0	0	0	0	0.088	0.0006	0	0	0.6781	0.0277	0	0	0	0.352	0.6781	0.6781	S	0
307	M	0.0002	0.0001	0	0	0	0	0	0.2564	0	0.0237	0.6915	0	0	0.012	0	0.0033	0.0069	0.0057	0	0	0.278	0.6915	0.6915	M	0
308	D	0.0083	0	0.9332	0.0047	0	0.0335	0	0	0	0	0	0	0	0	0	0.0159	0.0042	0	0	0	0.1116	0.9332	0.9332	D	0
309	R	0	0	0	0	0	0	0.0045	0	0	0	0	0	0	0	0.9942	0.0012	0	0	0	0	0.013	0.9942	0.9942	R	0
310	T	0.0012	0	0.0076	0	0	0.0066	0	0	0	0	0	0	0	0	0	0.4651	0.5194	0	0	0	0.2592	0.5194	0.5194	T	0
311	G	0	0	0.0085	0	0	0.971	0	0	0	0	0	0.0205	0	0	0	0	0	0	0	0	0.0498	0.971	0.971	G	0
312	Y	0	0.0019	0	0	0.1886	0	0.0161	0.0001	0	0.0002	0	0	0	0	0	0	0	0.0001	0.0034	0.7895	0.2015	0.7895	0.7895	Y	0
313	A	0.8281	0.0001	0	0	0	0	0	0.0035	0	0.0002	0	0	0	0	0	0.0335	0.003	0.1315	0	0	0.1931	0.8281	0.8281	A	0
314	R	0.0756	0.0246	0	0.0001	0	0	0.0001	0	0.0167	0.016	0.0047	0.0026	0	0.0076	0.8016	0.0289	0.0165	0	0	0.0048	0.2921	0.8016	0.8016	R	0
315	G	0.1128	0	0	0	0	0.8393	0	0.0072	0	0	0	0	0	0	0	0.0001	0.0404	0	0	0	0.1874	0.8393	0.8393	G	0
316	T	0.0002	0.0001	0	0	0	0	0	0.0014	0	0.0002	0.0012	0	0	0	0	0.0232	0.9683	0.0053	0	0	0.0564	0.9683	0.9683	T	0
317	L	0	0	0	0	0	0	0	0	0	0.9988	0	0	0	0	0	0	0	0.0012	0	0	0.0032	0.9988	0.9988	L	0
318	A	0.799	0	0	0	0	0.0061	0	0.0008	0	0	0	0	0	0	0	0.0642	0.1265	0.0032	0	0	0.2252	0.799	0.799	A	0
319	P	0.0462	0.0015	0.0002	0.0001	0.0005	0.0002	0.0026	0.0267	0.0001	0.0888	0.0132	0.0002	0.2211	0.0102	0.0679	0.131	0.175	0.2083	0	0.0061	0.6809	0.2211	0.2211	P	0
320	R	0.1198	0.0001	0.0334	0.0104	0.0001	0.0039	0.0095	0.0002	0.1354	0.0003	0.0001	0.0104	0.0002	0.0816	0.5636	0.0156	0.0048	0.0026	0	0.0081	0.4941	0.5636	0.5636	R	0
321	A	0.1983	0	0.0345	0.2921	0.0001	0.0083	0.0131	0.0108	0.0452	0.0082	0.0172	0.0004	0.2357	0.0683	0.0058	0.0132	0.0299	0.0126	0.0001	0.0061	0.6672	0.1983	0.2921	E	0.0937
322	G	0.0191	0	0.0021	0.0008	0	0.9208	0	0	0	0	0	0.0255	0	0	0.0004	0.0226	0.0085	0	0	0	0.1319	0.9208	0.9208	G	0
323	M	0.1208	0.0001	0	0	0.0007	0.0006	0	0.0207	0	0.5578	0.2316	0	0	0.0121	0.0012	0.0017	0.0011	0.0477	0	0.0038	0.4199	0.2316	0.5578	L	0.3261
324	T	0.0526	0.0001	0.0227	0.0574	0.0001	0.0009	0.0117	0.0078	0.0383	0.0305	0.0047	0.0088	0.0045	0.2118	0.1449	0.2947	0.0701	0.0358	0	0.0025	0.706	0.0701	0.2947	S	0.2247
325	A	0.9398	0	0	0	0	0.0246	0	0	0	0	0	0	0.0209	0	0	0.0041	0.0058	0.0047	0	0	0.1034	0.9398	0.9398	A	0
326	A	0.2246	0.0001	0.0247	0.0823	0.0002	0.0051	0.0005	0.0086	0.0199	0.0029	0.0001	0.0003	0.4983	0.0144	0.0039	0.0195	0.0297	0.0646	0.0001	0.0002	0.5323	0.2246	0.4983	P	0.2737
327	V	0.0123	0.0001	0	0	0	0.0012	0	0.1052	0	0.0268	0.0013	0	0.0098	0	0	0.0073	0.0335	0.8023	0	0	0.2599	0.8023	0.8023	V	0
328	P	0.0001	0	0.0001	0.0001	0	0.0001	0.0002	0.0001	0.0001	0.0009	0.0001	0.0001	0.9942	0.0002	0.0001	0.0032	0.0001	0.0001	0	0.0001	0.0154	0.9942	0.9942	P	0
329	T	0.4037	0.0039	0.0259	0.1241	0.0077	0.0228	0.0047	0.0136	0.0337	0.0219	0.0047	0.0152	0.0458	0.0239	0.0244	0.0788	0.1175	0.0186	0.0027	0.0063	0.7112	0.1175	0.4037	A	0.2862
330	P	0.0002	0.0001	0	0	0	0	0	0.0104	0	0.3277	0.1841	0	0.3502	0.0022	0.0088	0	0.0198	0.0964	0	0	0.4856	0.3502	0.3502	P	0
331	D	0.0001	0	0.788	0.0096	0	0.0006	0.0002	0.0004	0.0001	0.177	0.012	0.0001	0	0.0002	0.0089	0.0001	0.001	0.0014	0	0.0001	0.2226	0.788	0.788	D	0
332	K	0.0717	0.0069	0.2121	0.034	0.0138	0.0383	0.0125	0.0182	0.184	0.0319	0.0083	0.0163	0.1885	0.0278	0.027	0.0402	0.0262	0.0261	0.0049	0.0112	0.8061	0.184	0.2121	D	0.0281
333	P	0.0307	0.0001	0.0027	0.0007	0.0003	0.0006	0.0002	0.0829	0.0113	0.0279	0.0058	0.0004	0.5984	0.0004	0.157	0.0009	0.0063	0.073	0.0001	0.0002	0.4543	0.5984	0.5984	P	0
334	Q	0.0233	0	0.0001	0.1505	0	0	0.0223	0	0.0029	0.0046	0.008	0.0001	0.1132	0.0623	0.5326	0.0145	0.0172	0.0483	0	0	0.5253	0.0623	0.5326	R	0.4703
335	P	0.0082	0.0001	0.0004	0.0326	0.0002	0.0004	0.0041	0.0233	0.0106	0.0437	0.0071	0.0061	0.5656	0.0273	0.0136	0.0104	0.0064	0.0148	0.2249	0.0001	0.4938	0.5656	0.5656	P	0
336	L	0.0023	0.0001	0	0	0.0004	0	0	0.0402	0	0.9015	0.031	0	0	0	0.0088	0	0.0001	0.0152	0	0.0001	0.1529	0.9015	0.9015	L	0
337	E	0.0602	0.0001	0.021	0.0697	0.0001	0.0589	0.0001	0.0005	0.0002	0.0424	0.0001	0.0047	0.0001	0.0008	0.0215	0.233	0.4807	0.0058	0	0.0001	0.5283	0.0697	0.4807	T	0.411
338	M	0.0001	0	0.0001	0.0001	0.0011	0	0.0418	0	0.0001	0.0012	0.8915	0.0001	0.0166	0.0001	0.001	0.0386	0.0074	0	0	0.0001	0.165	0.8915	0.8915	M	0
339	E	0.2151	0	0.3021	0.0885	0.0001	0.0308	0.0008	0.0128	0.0151	0.0001	0.0574	0.032	0.0009	0.0422	0.041	0.0647	0.0795	0.0169	0	0.0001	0.7123	0.0885	0.3021	D	0.2136
340	D	0	0	0.9824	0.009	0	0.0028	0.0022	0	0	0	0	0	0	0.0001	0	0.0026	0.0006	0	0	0	0.0379	0.9824	0.9824	D	0
341	M	0.0002	0.0001	0.0463	0	0	0.0022	0	0.0057	0	0.0012	0.9222	0.0005	0	0	0	0.0001	0.0006	0.0208	0	0	0.1209	0.9222	0.9222	M	0
342	M	0.0081	0.0001	0.0009	0.0003	0.0001	0.0472	0.1119	0.0002	0.0045	0.0003	0.7147	0.0636	0.0012	0.0175	0.0002	0.0253	0.0013	0.0026	0.0001	0.0001	0.3609	0.7147	0.7147	M	0
343	G	0.0575	0.0001	0.027	0.0006	0.0003	0.8042	0.0235	0.0003	0.0012	0.0005	0.0284	0.001	0.0132	0.0026	0.001	0.0374	0.0005	0.0004	0.0001	0.0002	0.2894	0.8042	0.8042	G	0
344	D	0.1714	0.0004	0.5612	0.0155	0.0007	0.087	0.0059	0.0009	0.0021	0.0016	0.001	0.0497	0.001	0.0219	0.001	0.0717	0.0049	0.0012	0.0002	0.0006	0.4856	0.5612	0.5612	D	0
345	M	0.005	0.0002	0.0039	0.0005	0.0003	0.0071	0.0173	0.003	0.0051	0.0021	0.9379	0.0004	0.0045	0.0009	0.0011	0.0093	0.0005	0.0005	0.0001	0.0003	0.1249	0.9379	0.9379	M	0

346	T	0.2451	0.0002	0.0526	0.0076	0.0004	0.1628	0.0046	0.0004	0.025	0.0008	0.0166	0.0909	0.0742	0.0189	0.0019	0.2225	0.0681	0.0042	0.003	0.0004	0.6972	0.0681	0.2451	A	0.177
347	G	0.0902	0.0001	0.0313	0.006	0.0002	0.7639	0.0156	0.0001	0.0178	0.0002	0.0007	0.0164	0.0065	0.0008	0.0004	0.0426	0.0066	0.0002	0.0001	0.0002	0.3303	0.7639	0.7639	G	0
348	M	0.0244	0	0.0143	0.0094	0	0.0225	0.0001	0.0027	0.0343	0.0054	0.8146	0.0299	0.0065	0.0056	0.0069	0.0168	0.0024	0.0041	0	0.0001	0.3059	0.8146	0.8146	M	0
349	A	0.2395	0.001	0.0955	0.0171	0.0056	0.1381	0.0026	0.0043	0.0248	0.0085	0.2823	0.0024	0.0373	0.0022	0.0017	0.0718	0.031	0.0232	0.0005	0.0106	0.6913	0.2395	0.2823	M	0.0428
350	R	0.0479	0.0003	0.0206	0.0123	0.0006	0.0478	0.1066	0.0008	0.1445	0.0101	0.099	0.0147	0.011	0.3923	0.0394	0.0158	0.0324	0.0032	0.0002	0.0006	0.6804	0.0394	0.3923	Q	0.3528
351	A	0.2982	0.0006	0.0182	0.0027	0.0007	0.0561	0.003	0.0011	0.0261	0.0062	0.0475	0.0046	0.0245	0.0339	0.002	0.3217	0.0279	0.1245	0.0002	0.0005	0.6305	0.2982	0.3217	S	0.0235
352	H	0.0009	0	0.059	0.012	0	0.006	0.572	0	0.0857	0.0001	0.0269	0.0213	0	0.0161	0.1434	0.041	0.0155	0	0	0.0001	0.5038	0.572	0.572	H	0
353	H	0.0131	0	0.0224	0.0048	0.0084	0.0045	0.5014	0	0.0001	0.0006	0.0168	0.0011	0.4184	0.0016	0.0058	0.0011	0.0001	0	0	0.0001	0.3579	0.5014	0.5014	H	0
354	A	0.7009	0.0003	0.0667	0.0153	0.0005	0.0136	0.0001	0.0014	0.0048	0.0143	0.0201	0.0001	0.0875	0.0001	0.0001	0.0575	0.0047	0.0119	0	0.0001	0.3982	0.7009	0.7009	A	0
355	R	0.0581	0.0001	0.0457	0.0221	0.0001	0.0235	0.0073	0.0002	0.1543	0.0004	0.0018	0.0075	0.0357	0.0238	0.1595	0.4314	0.0222	0.0061	0	0.0002	0.6136	0.1595	0.4314	S	0.2718
356	T	0.0152	0.0002	0.0005	0.4239	0.0003	0.0006	0.0002	0.0004	0.0019	0.0107	0.0702	0.0005	0.0004	0.0003	0.0004	0.082	0.3858	0.0062	0.0001	0.0002	0.4374	0.3858	0.4239	E	0.0381
357	E	0.0259	0	0.1382	0.4534	0.0001	0.0136	0.0002	0.0002	0.0784	0.0008	0.0018	0.0559	0.0004	0.0614	0.0016	0.0303	0.1367	0.0009	0	0.0001	0.5802	0.4534	0.4534	E	0
358	Y	0.0068	0.0001	0.1229	0.0013	0.0289	0.1283	0.0872	0.0028	0.0186	0.0074	0.024	0.1895	0.0014	0.0093	0.0169	0.0191	0.0371	0.0018	0.0003	0.2962	0.7028	0.2962	0.2962	Y	0
359	G	0.0001	0	0.0874	0.0001	0	0.3747	0.0001	0	0.0001	0.0001	0	0.5119	0	0.0121	0.0001	0.0084	0.0047	0	0	0	0.3503	0.3747	0.5119	N	0.1372
360	P	0.0272	0	0	0	0.0853	0	0	0.0001	0	0.0048	0	0.0019	0.8403	0	0	0.015	0	0.021	0.0001	0.0042	0.221	0.8403	0.8403	P	0
361	S	0.0202	0	0.0078	0.0121	0.0103	0.1102	0.0337	0	0.0806	0.5681	0.0178	0.0189	0	0.0121	0.0001	0.1029	0.005	0	0	0.0001	0.5213	0.1029	0.5681	L	0.4652
362	V	0.0002	0.0001	0	0	0	0	0	0.021	0	0.0037	0.0088	0.0047	0	0	0	0.0641	0.0818	0.8153	0	0	0.2406	0.8153	0.8153	V	0
363	D	0.009	0	0.9662	0.0006	0	0.0001	0	0	0	0	0	0	0	0	0	0.0048	0.0191	0	0	0	0.0614	0.9662	0.9662	D	0
364	M	0.0198	0	0	0	0	0.0027	0	0	0	0	0.9586	0.0188	0	0	0	0	0	0	0	0	0.07	0.9586	0.9586	M	0
365	R	0.0001	0	0.0001	0.0001	0	0	0.0879	0.0068	0.0001	0.0068	0.0187	0.0001	0	0.601	0.2661	0.0001	0.0001	0.012	0	0	0.3584	0.2661	0.601	Q	0.3349
366	V	0.3715	0	0	0	0	0.0067	0	0	0	0	0	0	0.0128	0	0	0.015	0.2894	0.3043	0	0	0.4151	0.3043	0.3715	A	0.0672
367	N	0.0026	0	0.2933	0.0082	0	0	0.0065	0.0004	0.0001	0.0032	0.6275	0.0219	0.0022	0.0001	0.0029	0.0001	0.0154	0.0155	0	0.0001	0.3364	0.0219	0.6275	M	0.6056
368	T	0.0465	0.0001	0.0227	0.0002	0.0103	0.0005	0.0212	0.0111	0.0149	0.0017	0.2343	0.0421	0.0006	0.0157	0.0002	0.3533	0.2186	0.0031	0	0.0032	0.59	0.2186	0.3533	S	0.1347
369	P	0.0169	0.0001	0	0	0	0	0	0.0009	0	0.0025	0	0	0.7265	0	0	0	0.1941	0.0589	0	0	0.2705	0.7265	0.7265	P	0
370	R	0.0783	0	0	0	0	0.0077	0	0.0038	0.0354	0.0062	0.0166	0	0	0.0636	0.3241	0.1816	0.2108	0.0717	0	0	0.6161	0.3241	0.3241	R	0
371	T	0.092	0.0001	0.0021	0.0083	0.0003	0.0006	0.0273	0.0102	0.0006	0.0119	0.0856	0.0019	0.4782	0.0004	0.0068	0.0503	0.1799	0.0096	0.0001	0.0337	0.5718	0.1799	0.4782	P	0.2983
372	D	0.0002	0.0001	0.0045	0.0006	0.0001	0.0002	0.0002	0.0001	0.5278	0.0003	0.0162	0.3191	0.0001	0.0002	0.1109	0.0155	0.0033	0.0002	0	0.0001	0.3813	0.0045	0.5278	K	0.5234
373	L	0	0	0	0	0.0025	0	0	0.0073	0	0.9779	0	0	0	0.012	0	0	0	0	0	0.0001	0.0429	0.9779	0.9779	L	0
374	D	0.1177	0	0.7036	0.0152	0	0.0093	0.0093	0	0.0001	0.0001	0	0.0978	0.0001	0.0002	0.0001	0.0446	0.0001	0.0001	0	0.0018	0.3455	0.7036	0.7036	D	0
375	D	0.0007	0	0.9716	0.0156	0	0	0	0	0	0	0	0	0	0.012	0	0	0	0	0	0	0.0508	0.9716	0.9716	D	0
376	P	0.0253	0	0	0	0	0	0	0	0	0.0006	0	0	0.9715	0	0	0	0.0025	0	0	0	0.0472	0.9715	0.9715	P	0
377	G	0	0	0	0	0	0.9987	0	0	0	0	0	0	0	0	0	0.0013	0	0	0	0	0.0035	0.9987	0.9987	G	0
378	V	0.0003	0.0001	0.0207	0	0.0089	0	0	0.5736	0	0.1528	0.0513	0.003	0.0191	0	0	0.0016	0.0174	0.151	0	0.0001	0.4492	0.151	0.5736	I	0.4227
379	G	0	0	0	0	0	0.8782	0	0	0	0	0	0.1137	0	0	0.008	0	0	0	0	0	0.1337	0.8782	0.8782	G	0
380	L	0	0	0	0	0	0	0	0	0	0.9988	0	0	0.0011	0	0	0	0	0	0	0	0.0031	0.9988	0.9988	L	0
381	R	0	0.0016	0.0188	0	0	0.0092	0.0009	0	0	0	0.0026	0	0	0	0.9666	0	0	0	0	0	0.062	0.9666	0.9666	R	0
382	D	0.0015	0	0.426	0.0391	0	0.0766	0.0039	0	0.0244	0.0001	0	0.4171	0.0001	0.0053	0.0001	0.0053	0.0002	0.0001	0	0.0001	0.4127	0.426	0.426	D	0
383	N	0.0001	0	0.0088	0.0001	0	0	0.0053	0.0722	0.0124	0.0001	0.0034	0.8356	0	0.0064	0.0372	0.0172	0.0001	0.0008	0	0.0001	0.2399	0.8356	0.8356	N	0
384	G	0.0136	0	0.0541	0.0006	0	0.8527	0.0001	0	0.0119	0.0001	0	0.0406	0.0001	0.0001	0.0031	0.0076	0.0152	0.0001	0	0	0.2213	0.8527	0.8527	G	0
385	R	0	0	0	0	0	0	0.0376	0	0	0	0	0	0	0	0.9482	0	0	0	0.0141	0	0.0783	0.9482	0.9482	R	0
386	R	0.0021	0	0.0267	0.0292	0	0	0.0022	0	0.2312	0.0001	0	0.0025	0	0.005	0.6949	0.0001	0.0057	0	0	0.0001	0.298	0.6949	0.6949	R	0
387	V	0.0031	0.0001	0	0	0	0	0	0.0012	0	0.0002	0	0	0	0	0	0.0021	0.0036	0.9895	0	0	0.0249	0.9895	0.9895	V	0
388	L	0.0033	0	0	0	0	0	0	0	0	0.9967	0	0	0	0	0	0	0	0	0	0	0.0075	0.9967	0.9967	L	0
389	T	0.0086	0.0021	0	0	0	0	0	0.0006	0	0.0002	0	0.0564	0	0	0.0237	0.0187	0.8661	0.0233	0	0	0.2003	0.8661	0.8661	T	0
390	Y	0	0.0011	0	0	0	0	0	0	0	0.0808	0	0	0	0	0	0	0	0	0	0.9181	0.0967	0.9181	0.9181	Y	0
391	A	0.7502	0	0.0167	0.0001	0	0.0325	0.001	0	0	0.0025	0	0.0046	0	0.0111	0.0011	0.1633	0.0166	0	0	0	0.2893	0.7502	0.7502	A	0
392	D	0.0021	0	0.8889	0.0001	0	0	0.0029	0	0.0001	0.0001	0.0191	0.0005	0	0.0781	0.0056	0.0001	0.0001	0.0024	0	0.0001	0.154	0.8889	0.8889	D	0
393	L	0	0	0	0	0	0	0	0.0097	0	0.9607	0.0295	0	0	0	0	0	0	0	0	0	0.0628	0.9607	0.9607	L	0
394	H	0.0001	0.0005	0.0001	0.0142	0	0	0.2217	0	0.1441	0.0001	0	0.0029	0	0.0044	0.5836	0.0046	0.0033	0.0039	0	0.0164	0.3901	0.2217	0.5836	R	0.3619
395	T	0.0192	0	0	0	0	0.0003	0	0	0	0	0	0.024	0	0	0	0.7563	0.2	0	0	0	0.2346	0.2	0.7563	S	0.5564
396	V	0.0404	0.0001	0	0	0	0	0.0034	0.1416	0	0.1218	0.0057	0.0004	0.0294	0	0.1285	0.0072	0.3933	0.1282	0	0	0.5839	0.1282	0.3933	T	0.2652
397	G	0.0001	0.0063	0.076	0.0166	0.5829	0.227	0.0146	0	0.0121	0.0007	0	0.0001	0	0.0044	0.0157	0.0001	0.0057	0.0047	0	0.0331	0.4429	0.227	0.5829	F	0.3559
398	G	0.1503	0.0001	0.0646	0.295	0.0002	0.226	0.0035	0.0196	0.0006	0.0114	0.0115	0.0118	0.1328	0.026	0.0005	0.0213	0.0117	0.0095	0.0001	0.0035	0.6616	0.226	0.295	E	0.069
399	P	0.0651	0.0001	0.5952	0.0275	0.0088	0.0072	0.0054	0.0292	0.0004	0.0153	0.0026	0.0035	0.1265	0.0098	0.0003	0.0548	0.0385	0.0098	0	0.0001	0.5131	0.1265	0.5952	D	0.4687
400	L	0.0268	0.0001	0.0048	0.0055	0.0003	0.0007	0.0116	0.1507	0.0067	0.0876	0.0164	0.0203	0.6046	0.0004	0.017	0.0056	0.0357	0.0047	0.0001	0.0003	0.482	0.0876	0.6046	P	0.517
401	D	0.001	0.0001	0.8232	0.0981	0.0002	0.0006	0.0003	0.0003	0.0005	0.0004	0.0039	0.0502	0.0004	0.0004	0.005	0.0008	0.0005	0.0004	0.0001	0.0137	0.2304	0.8232	0.8232	D	0
402	P	0.0404	0.0002	0.0339	0.0055	0.0003	0.5901	0.009	0.009	0.0246	0.006	0.0002	0.0301	0.0618	0.097	0.0167	0.028	0.0336	0.0019	0.0001	0.0115	0.5498	0.0618	0.5901	G	0.5284
403	R	0.0006	0.0002	0.0004	0.0043	0.0003	0.0006	0.0003	0.0004	0.0233	0.0148	0.0002	0.0004	0.0118	0.0265	0.9113	0.0031	0.0005	0.0005	0.0001	0.0003	0.1543	0.9113	0.9113	R	0
404	D	0.0477	0.0001	0.1456	0.5595	0.0002	0.0142	0.0079	0.0059	0.08	0.0013	0.0199	0.0006	0.0473	0.0223	0.0009	0.0119	0.0184	0.0099	0.0001	0.0062	0.5392	0.1456	0.5595	E	0.414
405	G	0.0122	0.0001	0.0002	0.0003	0.0001	0.0027	0.0002	0.0002	0.0002	0.0003	0.0001	0.0002	0.9745	0.0003	0.0002	0.004	0.0002	0.0036	0.0001	0.0001	0.0541	0.0027	0.9745	P	0.9718
406	E	0.0185	0.0001	0.0546	0.0488	0.0001	0.3503	0.0001	0.0002	0.0039	0.0002	0.0028	0.0123	0.0001	0.0111	0.0037	0.3443	0.1452	0.0037	0	0.0001	0.5292	0.0488	0.3503	G	0.3016
407	R	0.0206	0	0.0041	0	0	0	0	0	0.0146	0	0	0	0	0	0.9606	0	0	0	0	0	0.0681	0.9606	0.9606	R	0
408	E	0.0035	0	0.0813	0.3444	0	0	0.0001	0	0	0.0001	0	0.0066	0	0.0001	0.0001	0.0019	0.5616	0	0	0	0.3226	0.3444	0.5616	T	0.2173
409	I	0.0002	0.0001	0	0	0	0	0	0.8141	0	0.0894	0.0163	0	0	0	0	0	0.0002	0.0794	0	0	0.2197	0.8141	0.8141	I	0
410	E	0.0016	0	0.0009	0.9319	0	0	0.0001	0.0047	0.0009	0.0001	0	0.0001	0	0.0163	0.0001	0.0001	0.0131	0.0299	0	0	0.1166	0.9319	0.9319	E	0
411	L	0	0	0	0	0.0003	0	0	0.046	0	0.9218	0.0193	0	0	0	0	0	0	0.0001	0.0001	0.0122	0.118	0.9218	0.9218	L	0
412	H	0	0	0	0	0	0	0.9837	0	0	0	0	0	0	0	0.0162	0	0	0	0	0	0.0279	0.9837	0.9837	H	0
413	L	0	0	0	0	0	0	0	0	0	0.9904	0	0	0	0	0.0008	0	0	0.0088	0	0	0.0192	0.9904	0.9904	L	0
414	T	0	0	0	0	0	0	0	0	0	0	0	0	0	0	0	0	0.9999	0	0	0	0.0003	0.9999	0.9999	T	0
415	G	0	0	0	0	0	0.9988	0	0	0	0	0	0	0	0	0	0.0012	0	0	0	0	0.0033	0.9988	0.9988	G	0
416	N	0.0018	0	0.0001	0.0001	0	0	0.5824	0	0.0001	0.0001	0	0.4057	0	0.0013	0.0001	0.0076	0.0001	0	0	0.0006	0.2497	0.4057	0.5824	H	0.1767
417	M	0	0	0	0	0	0	0	0	0	0	0.9999	0	0	0	0	0	0	0	0	0	0.0003	0.9999	0.9999	M	0
418	E	0.0112	0.0003	0.0241	0.8907	0.0007	0.0034	0.005	0.0009	0.001	0.0015	0.0004	0.007	0.0009	0.0196	0.0009	0.0294	0.001	0.0011	0.0002	0.0006	0.191	0.8907	0.8907	E	0
419	R	0.0013	0.0003	0.0009	0.0011	0.0007	0.0012	0.0017	0.0009	0.499	0.0015	0.0004	0.0008	0.0009	0.0008	0.4843	0.0012	0.001	0.0011	0.0002	0.0006	0.2715	0.4843	0.499	K	0.0147
420	Y	0.0013	0.0003	0.0009	0.0011	0.6581	0.0012	0.0005	0.0009	0.001	0.0016	0.0004	0.0008	0.0009	0.0008	0.0009	0.0012	0.001	0.0011	0.0123	0.3134	0.2686	0.3134	0.6581	F	0.3447
421	T	0.5017	0.0007	0.0009	0.0012	0.0008	0.0019	0.0005	0.1223	0.0011	0.0021	0.1396	0.0008	0.0009	0.0008	0.0025	0.1259	0.0503	0.0453	0.0003	0.0006	0.5106	0.0503	0.5017	A	0.4514
422	W	0.0013	0.0003	0.0009	0.0011	0.0007	0.0012	0.0005	0.0009	0.001	0.0015	0.0004	0.0008	0.0009	0.0008	0.0009	0.0012	0.001	0.0011	0.9827	0.0006	0.0457	0.9827	0.9827	W	0
423	S	0.0815	0.0003	0.0009	0.0011	0.0007	0.0856	0.0005	0.0009	0.001	0.0015	0.0004	0.0008	0.0009	0.0008	0.0009	0.8169	0.0032	0.0011	0.0002	0.0006	0.2298	0.8169	0.8169	S	0
424	I	0.0014	0.0004	0.0009	0.0011	0.8307	0.0012	0.0005	0.0682	0.001	0.0727	0.0133	0.0008	0.0009	0.0008	0.0009	0.0012	0.001	0.0012	0.0003	0.0016	0.2301	0.0682	0.8307	F	0.7625
425	D	0.0015	0.0004	0.7087	0.0018	0.0007	0.0023	0.0006	0.0009	0.0012	0.0016	0.0004	0.2714	0.001	0.0009	0.001	0.0025	0.0013	0.0012	0.0002	0.0006	0.2438	0.7087	0.7087	D	0
426	G	0.0016	0.0004	0.0012	0.0012	0.0007	0.9816	0.0006	0.0009	0.0012	0.0016	0.0004	0.0011	0.001	0.0009	0.001	0.0015	0.0011	0.0012	0.0003	0.0006	0.0482	0.9816	0.9816	G	0
427	V	0.0016	0.0004	0.0009	0.0044	0.0009	0.0012	0.0004	0.3638	0.0321	0.0949	0.0121	0.0007	0.0009	0.0522	0.0125	0.0012	0.0051	0.4138	0.0003	0.0006	0.4817	0.4138	0.4138	V	0
428	E	0.0013	0.0003	0.0009	0.0974	0.0007	0.0012	0.0005	0.0009	0.7409	0.0015	0.0482	0.0008	0.0696	0.0008	0.005	0.008	0.0199	0.0011	0.0002	0.0006	0.3339	0.0974	0.7409	K	0.6435
429	F	0.0047	0.0003	0.0009	0.0011	0.9112	0.0012	0.0005	0.0009	0.001	0.0118	0.003	0.0008	0.0009	0.0008	0.0009	0.0016	0.001	0.0011	0.0002	0.0559	0.1449	0.9112	0.9112	F	0
430	G	0.1292	0.0012	0.0024	0.0099	0.0007	0.0755	0.0085	0.003	0.0518	0.0016	0.0078	0.0134	0.0023	0.0053	0.0059	0.672	0.0072	0.0013	0.0002	0.0006	0.4142	0.0755	0.672	S	0.5965
431	R	0.0155	0.0004	0.7169	0.1207	0.0007	0.0144	0.0045	0.0009	0.0587	0.0016	0.0004	0.0065	0.001	0.0287	0.0107	0.0151	0.0013	0.0012	0.0002	0.0006	0.372	0.0107	0.7169	D	0.7062
432	S	0.8092	0.0007	0.0009	0.0012	0.0008	0.0027	0.0005	0.0013	0.0098	0.0021	0.0006	0.0008	0.0009	0.0008	0.001	0.1491	0.0014	0.0154	0.0003	0.0006	0.2256	0.1491	0.8092	A	0.66
433	T	0.0899	0.0004	0.0177	0.4534	0.0007	0.0098	0.0006	0.001	0.2134	0.0018	0.0005	0.0012	0.0131	0.025	0.0073	0.0153	0.138	0.0098	0.0003	0.0006	0.5472	0.138	0.4534	E	0.3154
434	P	0.0025	0.0003	0.0009	0.0011	0.0007	0.0012	0.0005	0.0009	0.001	0.0016	0.0004	0.0008	0.982	0.0008	0.0009	0.0012	0.001	0.0011	0.0002	0.0006	0.047	0.982	0.982	P	0
435	V	0.0015	0.0004	0.0009	0.001	0.001	0.0012	0.0004	0.1463	0.001	0.5189	0.0039	0.0007	0.0009	0.0007	0.0009	0.0012	0.0011	0.3172	0.0003	0.0006	0.3682	0.3172	0.5189	L	0.2016
436	H	0.015	0.0011	0.0018	0.0315	0.0012	0.0023	0.1878	0.0059	0.0352	0.0646	0.0602	0.0814	0.0016	0.0699	0.3691	0.0169	0.0194	0.031	0.0004	0.0038	0.6899	0.1878	0.3691	R	0.1813
437	F	0.0023	0.0006	0.0014	0.0017	0.2071	0.002	0.0006	0.099	0.0016	0.6452	0.0258	0.0012	0.0014	0.0012	0.0014	0.0019	0.0017	0.0023	0.0004	0.001	0.3605	0.2071	0.6452	L	0.4381
438	R	0.0151	0.0006	0.0018	0.0149	0.0012	0.0052	0.0255	0.0028	0.5044	0.0029	0.0013	0.0656	0.0188	0.0119	0.1375	0.0035	0.1696	0.0021	0.0004	0.015	0.5462	0.1375	0.5044	K	0.3669
439	H	0.0073	0.0007	0.0017	0.0042	0.0435	0.0023	0.0573	0.0017	0.0121	0.0421	0.0008	0.0015	0.0866	0.0154	0.0136	0.0282	0.0119	0.0021	0.0007	0.6661	0.4606	0.0573	0.6661	Y	0.6088
440	N	0.0029	0.0006	0.0033	0.0022	0.0012	0.8706	0.0048	0.0016	0.0046	0.0027	0.0007	0.0888	0.0017	0.0014	0.0018	0.0058	0.0019	0.002	0.0004	0.001	0.1919	0.0888	0.8706	G	0.7818
441	E	0.0029	0.0008	0.002	0.9429	0.0015	0.0028	0.001	0.002	0.0064	0.0035	0.0009	0.0017	0.0019	0.0186	0.002	0.0027	0.0023	0.0025	0.0005	0.0012	0.1203	0.9429	0.9429	E	0
442	R	0.0029	0.0008	0.002	0.0024	0.0015	0.0028	0.0085	0.002	0.0023	0.0035	0.0009	0.0017	0.0019	0.0017	0.9324	0.0027	0.011	0.0173	0.0005	0.0012	0.1386	0.9324	0.9324	R	0
443	L	0.0031	0.0008	0.0019	0.0023	0.0023	0.0028	0.0009	0.0732	0.0022	0.3792	0.0192	0.0016	0.0019	0.0016	0.0019	0.0026	0.0023	0.4982	0.0005	0.0013	0.3903	0.3792	0.4982	V	0.119
444	R	0.0029	0.0014	0.002	0.0024	0.0015	0.0028	0.001	0.002	0.0023	0.0042	0.0009	0.0017	0.0019	0.0017	0.9621	0.0027	0.0023	0.0025	0.0005	0.0012	0.0901	0.9621	0.9621	R	0
445	V	0.0032	0.0008	0.0019	0.0023	0.0739	0.0028	0.0009	0.5772	0.0022	0.1062	0.005	0.0016	0.0019	0.0015	0.0019	0.0026	0.0023	0.2097	0.0005	0.0013	0.4257	0.2097	0.5772	I	0.3675
446	V	0.0036	0.0008	0.0021	0.0025	0.0019	0.0029	0.0009	0.068	0.0023	0.0046	0.001	0.0019	0.002	0.0016	0.0108	0.007	0.3342	0.5499	0.0005	0.0013	0.3821	0.5499	0.5499	V	0
447	F	0.003	0.0008	0.002	0.0023	0.0556	0.0028	0.0009	0.0027	0.0022	0.8999	0.0012	0.0016	0.0019	0.0016	0.0019	0.0026	0.0023	0.0029	0.0006	0.0112	0.1705	0.0556	0.8999	L	0.8443
448	V	0.0065	0.0049	0.002	0.0024	0.0016	0.0028	0.023	0.0876	0.0022	0.0038	0.001	0.0017	0.0019	0.0317	0.002	0.0026	0.0023	0.8183	0.0005	0.0013	0.2684	0.8183	0.8183	V	0
449	N	0.003	0.0008	0.0025	0.0024	0.0016	0.0031	0.0011	0.002	0.0024	0.0035	0.0009	0.9611	0.0019	0.0016	0.002	0.0031	0.0025	0.0025	0.0005	0.0013	0.0919	0.9611	0.9611	N	0
450	D	0.0031	0.0008	0.9538	0.0032	0.0015	0.0031	0.001	0.002	0.0025	0.0035	0.0009	0.0087	0.0021	0.0017	0.0021	0.0031	0.0025	0.0025	0.0005	0.0013	0.1048	0.9538	0.9538	D	0
451	T	0.0033	0.0008	0.0022	0.0025	0.0016	0.003	0.0009	0.0021	0.0024	0.0035	0.001	0.0025	0.002	0.0016	0.002	0.0116	0.9525	0.0027	0.0005	0.0013	0.1062	0.9525	0.9525	T	0
452	M	0.0029	0.0008	0.002	0.0024	0.0015	0.0028	0.001	0.002	0.0023	0.0035	0.9522	0.0017	0.0019	0.0017	0.002	0.0027	0.0125	0.0025	0.0005	0.0012	0.1063	0.9522	0.9522	M	0
453	M	0.0029	0.0008	0.002	0.0024	0.0015	0.0028	0.001	0.002	0.0023	0.0035	0.9625	0.0017	0.0019	0.0017	0.002	0.0027	0.0023	0.0025	0.0005	0.0012	0.0894	0.9625	0.9625	M	0
454	T	0.039	0.0008	0.0022	0.0455	0.0016	0.0053	0.04	0.0123	0.0024	0.0035	0.0204	0.2134	0.0078	0.0016	0.002	0.0667	0.5309	0.0027	0.0005	0.0013	0.5196	0.5309	0.5309	T	0
455	H	0.0029	0.0008	0.002	0.0024	0.0015	0.0028	0.9625	0.002	0.0023	0.0035	0.0009	0.0017	0.0019	0.0017	0.002	0.0027	0.0023	0.0025	0.0005	0.0012	0.0892	0.9625	0.9625	H	0
456	P	0.0038	0.0008	0.0024	0.0029	0.0017	0.0032	0.001	0.0023	0.0027	0.0039	0.001	0.0019	0.958	0.0018	0.0023	0.0032	0.0026	0.0029	0.0006	0.0013	0.0981	0.958	0.958	P	0
457	M	0.0029	0.0008	0.002	0.0024	0.0015	0.0028	0.001	0.741	0.0023	0.0122	0.2053	0.0017	0.0019	0.0017	0.002	0.0027	0.0023	0.0118	0.0005	0.0012	0.2796	0.2053	0.741	I	0.5357
458	H	0.0029	0.0008	0.002	0.0024	0.0015	0.0028	0.9559	0.002	0.0023	0.0035	0.0009	0.0017	0.0019	0.0083	0.002	0.0027	0.0023	0.0025	0.0005	0.0012	0.101	0.9559	0.9559	H	0
459	L	0.003	0.0008	0.002	0.0023	0.0018	0.0028	0.0009	0.0026	0.0022	0.9363	0.022	0.0016	0.0019	0.0016	0.0019	0.0026	0.0023	0.0029	0.0006	0.0078	0.1308	0.9363	0.9363	L	0
460	H	0.0029	0.0008	0.002	0.0024	0.0015	0.0028	0.9625	0.002	0.0023	0.0035	0.0009	0.0017	0.0019	0.0017	0.002	0.0027	0.0023	0.0025	0.0005	0.0012	0.0892	0.9625	0.9625	H	0
461	G	0.0024	0.0005	0.0018	0.0017	0.001	0.9744	0.0007	0.0012	0.0017	0.0021	0.0006	0.0017	0.0013	0.0011	0.0014	0.0022	0.0015	0.0015	0.0003	0.0008	0.064	0.9744	0.9744	G	0

462	M	0.0021	0.0005	0.0014	0.0017	0.0131	0.0019	0.0007	0.0082	0.0016	0.1267	0.8285	0.0012	0.0013	0.0012	0.0014	0.0019	0.0016	0.0039	0.0004	0.0009	0.2218	0.8285	0.8285	M	0
463	W	0.0021	0.0005	0.0014	0.0017	0.0127	0.0019	0.0007	0.0014	0.0016	0.0144	0.0007	0.0012	0.0013	0.0012	0.0014	0.0019	0.0016	0.0017	0.9431	0.0075	0.1181	0.9431	0.9431	W	0
464	S	0.0026	0.0045	0.0017	0.0021	0.0133	0.0229	0.0009	0.0017	0.0051	0.003	0.0206	0.0032	0.0017	0.0015	0.0017	0.9078	0.002	0.0022	0.0005	0.0011	0.1747	0.9078	0.9078	S	0
465	E	0.0027	0.0007	0.7537	0.1926	0.0013	0.0027	0.0009	0.0018	0.0022	0.003	0.0008	0.0029	0.0018	0.0015	0.0018	0.0147	0.0022	0.0022	0.0005	0.0098	0.2733	0.1926	0.7537	D	0.5612
466	L	0.0027	0.0007	0.0017	0.002	0.0017	0.0024	0.0008	0.0208	0.0019	0.9099	0.0119	0.0014	0.0017	0.0063	0.0017	0.0023	0.002	0.0265	0.0005	0.0011	0.1679	0.9099	0.9099	L	0
467	E	0.0026	0.0096	0.0085	0.8685	0.0013	0.0024	0.0009	0.0017	0.002	0.003	0.0008	0.0015	0.0017	0.0015	0.086	0.0023	0.002	0.0022	0.0005	0.0011	0.1971	0.8685	0.8685	E	0
468	S	0.0302	0.0007	0.6148	0.0029	0.0013	0.0027	0.0009	0.0018	0.0022	0.0107	0.0365	0.0923	0.0018	0.0015	0.0018	0.0994	0.0882	0.0085	0.0005	0.0011	0.4675	0.0994	0.6148	D	0.5154
469	P	0.0567	0.0007	0.0243	0.5591	0.0015	0.0883	0.0009	0.002	0.003	0.0034	0.0009	0.0017	0.2387	0.0074	0.002	0.0029	0.0023	0.0026	0.0005	0.0012	0.4434	0.2387	0.5591	E	0.3205
470	D	0.012	0.0007	0.2072	0.058	0.0014	0.0065	0.1187	0.0018	0.0099	0.003	0.0008	0.356	0.0017	0.1636	0.0018	0.0452	0.0077	0.0022	0.0005	0.0011	0.6053	0.2072	0.356	N	0.1488
471	G	0.0176	0.0007	0.0214	0.0025	0.0014	0.895	0.001	0.0018	0.0024	0.0031	0.0009	0.02	0.0075	0.009	0.0025	0.007	0.0022	0.0023	0.0005	0.0012	0.1953	0.895	0.895	G	0
472	G	0.046	0.0007	0.0993	0.1096	0.0014	0.035	0.0085	0.0019	0.0512	0.0033	0.0156	0.305	0.0018	0.2194	0.0766	0.0073	0.0086	0.0071	0.0005	0.0012	0.6877	0.035	0.305	N	0.27
473	F	0.0149	0.0008	0.002	0.0023	0.8742	0.0027	0.0105	0.002	0.0022	0.028	0.001	0.0017	0.0018	0.0016	0.0068	0.003	0.0022	0.0065	0.0008	0.035	0.2209	0.8742	0.8742	F	0
474	Q	0.016	0.001	0.0026	0.0033	0.0022	0.0037	0.0111	0.029	0.0414	0.2496	0.2291	0.0022	0.0025	0.3571	0.0309	0.0035	0.003	0.0093	0.0007	0.0017	0.571	0.3571	0.3571	Q	0
475	V	0.0202	0.0007	0.0018	0.0022	0.0014	0.0026	0.0009	0.0026	0.0021	0.0327	0.0009	0.0016	0.0606	0.0015	0.0018	0.0024	0.0021	0.8602	0.0005	0.0011	0.2191	0.8602	0.8602	V	0
476	R	0.0027	0.0007	0.0018	0.0022	0.0082	0.0026	0.0009	0.0018	0.0165	0.0152	0.0009	0.0016	0.0018	0.0015	0.9332	0.0024	0.0021	0.0023	0.0005	0.0011	0.1352	0.9332	0.9332	R	0
477	K	0.0027	0.0007	0.0018	0.0022	0.0014	0.0026	0.0009	0.0018	0.9285	0.0032	0.0009	0.0016	0.0018	0.0015	0.0399	0.0024	0.0021	0.0023	0.0005	0.0011	0.1319	0.9285	0.9285	K	0
478	H	0.0027	0.0007	0.0018	0.0022	0.0014	0.0026	0.9642	0.0018	0.0021	0.0032	0.0009	0.0022	0.0018	0.0015	0.0025	0.0024	0.0021	0.0023	0.0005	0.0011	0.0858	0.9642	0.9642	H	0
479	T	0.003	0.0008	0.002	0.0023	0.0014	0.0027	0.0009	0.0027	0.0022	0.0033	0.0009	0.0026	0.0019	0.0015	0.0019	0.0082	0.9576	0.0025	0.0005	0.0012	0.0974	0.9576	0.9576	T	0
480	I	0.003	0.0008	0.0019	0.0022	0.0034	0.0027	0.0008	0.8031	0.0021	0.0361	0.0118	0.0015	0.0019	0.0015	0.0018	0.0025	0.0023	0.1188	0.0005	0.0012	0.2631	0.8031	0.8031	I	0
481	A	0.0218	0.0008	0.5752	0.0033	0.0016	0.0032	0.0011	0.0303	0.0025	0.0218	0.0498	0.1375	0.0398	0.0018	0.0021	0.0418	0.0196	0.0442	0.0005	0.0013	0.5346	0.0218	0.5752	D	0.5533
482	V	0.003	0.0008	0.002	0.0025	0.0015	0.0029	0.001	0.0392	0.0023	0.0083	0.5056	0.0017	0.002	0.0017	0.002	0.0027	0.0023	0.4166	0.0006	0.0013	0.3553	0.4166	0.5056	M	0.089
483	Q	0.0039	0.0008	0.0025	0.003	0.0018	0.0034	0.0126	0.0024	0.044	0.0041	0.001	0.0066	0.5901	0.3102	0.0024	0.0034	0.0028	0.003	0.0006	0.0014	0.3725	0.3102	0.5901	P	0.2799
484	P	0.0039	0.0008	0.0025	0.003	0.0018	0.0034	0.0011	0.0024	0.0028	0.0059	0.001	0.002	0.9485	0.0019	0.0033	0.0079	0.0028	0.0031	0.0006	0.0014	0.1152	0.9485	0.9485	P	0
485	A	0.285	0.0009	0.0029	0.003	0.0018	0.6629	0.0012	0.0023	0.003	0.004	0.0011	0.0027	0.0024	0.002	0.0025	0.0049	0.0106	0.0047	0.0006	0.0015	0.3081	0.285	0.6629	G	0.3779
486	Q	0.0658	0.0016	0.0024	0.0442	0.0017	0.0033	0.0073	0.0024	0.0106	0.004	0.0011	0.0021	0.0027	0.278	0.0023	0.3621	0.2033	0.003	0.0006	0.0014	0.5412	0.278	0.3621	S	0.084
487	R	0.0041	0.0013	0.0027	0.0035	0.0018	0.0035	0.0013	0.0067	0.3664	0.0043	0.0042	0.0023	0.0024	0.0437	0.5392	0.0036	0.0036	0.0031	0.0006	0.0015	0.3762	0.5392	0.5392	R	0
488	V	0.0036	0.0009	0.0024	0.0028	0.0019	0.0033	0.0011	0.2068	0.0027	0.0262	0.0012	0.002	0.0023	0.0019	0.583	0.0032	0.0027	0.1497	0.0006	0.0015	0.4098	0.1497	0.583	R	0.4333
489	S	0.0298	0.0018	0.0026	0.003	0.0019	0.0196	0.0011	0.0025	0.0028	0.0042	0.0011	0.0033	0.0024	0.002	0.0024	0.8388	0.0753	0.0032	0.0006	0.0015	0.248	0.8388	0.8388	S	0
490	F	0.004	0.001	0.0027	0.0031	0.1717	0.0037	0.0016	0.0027	0.003	0.0048	0.0114	0.0023	0.0025	0.0021	0.0028	0.0044	0.003	0.0121	0.0043	0.7567	0.2986	0.1717	0.7567	Y	0.585
491	L	0.0692	0.001	0.0957	0.0031	0.0019	0.0123	0.0013	0.0026	0.003	0.1113	0.0012	0.0022	0.0025	0.0021	0.6667	0.0034	0.0029	0.0152	0.0007	0.0016	0.4078	0.1113	0.6667	R	0.5554
492	V	0.0038	0.001	0.0026	0.0031	0.0139	0.0036	0.0013	0.0031	0.0029	0.0101	0.0012	0.0022	0.0025	0.0021	0.0025	0.0034	0.0166	0.922	0.0007	0.0016	0.1576	0.922	0.922	V	0
493	T	0.0041	0.0011	0.0027	0.0031	0.002	0.0037	0.0036	0.0027	0.0252	0.0045	0.0012	0.0199	0.0026	0.0021	0.0558	0.0921	0.7592	0.0034	0.0007	0.0103	0.3426	0.7592	0.7592	T	0
494	A	0.8014	0.0013	0.0025	0.0031	0.0021	0.0915	0.0012	0.003	0.0029	0.005	0.0014	0.0021	0.0196	0.0021	0.0026	0.0041	0.0033	0.0486	0.0007	0.0017	0.2839	0.8014	0.8014	A	0
495	D	0.0049	0.0011	0.8301	0.092	0.0022	0.0043	0.015	0.0029	0.0034	0.0049	0.0013	0.0161	0.0029	0.0024	0.0029	0.0042	0.0034	0.0036	0.0008	0.0018	0.2576	0.8301	0.8301	D	0
496	A	0.8237	0.0017	0.0172	0.0155	0.0028	0.0086	0.0016	0.0091	0.0041	0.0067	0.0018	0.0539	0.0035	0.0067	0.0035	0.0054	0.0205	0.0102	0.001	0.0023	0.2969	0.8237	0.8237	A	0
497	L	0.017	0.0027	0.0064	0.0291	0.0117	0.0094	0.0029	0.0243	0.0302	0.6533	0.0141	0.0054	0.1109	0.0054	0.0263	0.0088	0.0077	0.0276	0.0023	0.0044	0.494	0.6533	0.6533	L	0
498	G	0.0049	0.0011	0.0035	0.0037	0.0022	0.9433	0.0014	0.0029	0.0036	0.005	0.0013	0.0031	0.003	0.0024	0.0031	0.0058	0.0034	0.0036	0.0008	0.0018	0.1265	0.9433	0.9433	G	0
499	R	0.0129	0.0014	0.0029	0.0036	0.0022	0.0173	0.0352	0.0029	0.0036	0.005	0.0013	0.0081	0.0028	0.0161	0.8364	0.0342	0.0081	0.0036	0.0008	0.0018	0.2799	0.8364	0.8364	R	0
500	W	0.0041	0.0011	0.0028	0.0034	0.0021	0.0039	0.0014	0.0028	0.0032	0.0049	0.0013	0.0024	0.0027	0.0023	0.0028	0.0037	0.0032	0.0035	0.9466	0.0017	0.1209	0.9466	0.9466	W	0
501	A	0.941	0.0014	0.0028	0.0034	0.0023	0.0046	0.0013	0.0032	0.0032	0.0054	0.0015	0.0024	0.0028	0.0023	0.0028	0.0044	0.0083	0.0045	0.0008	0.0018	0.1301	0.941	0.941	A	0
502	W	0.0047	0.0012	0.0031	0.0037	0.1128	0.0044	0.0018	0.0032	0.0035	0.0056	0.0015	0.0027	0.003	0.0025	0.0032	0.0042	0.0035	0.0039	0.1532	0.6786	0.3707	0.1532	0.6786	Y	0.5254
503	H	0.0043	0.0011	0.0029	0.0035	0.0022	0.0041	0.9456	0.0029	0.0033	0.005	0.0013	0.0025	0.0028	0.0024	0.0029	0.0038	0.0033	0.0036	0.0008	0.0018	0.1227	0.9456	0.9456	H	0
504	C	0.0043	0.9453	0.0029	0.0035	0.0022	0.0041	0.0014	0.0029	0.0033	0.005	0.0013	0.0025	0.0028	0.0024	0.0029	0.0038	0.0033	0.0036	0.0008	0.0018	0.1233	0.9453	0.9453	C	0
505	H	0.0043	0.0011	0.0029	0.0035	0.0022	0.0041	0.9456	0.0029	0.0033	0.005	0.0013	0.0025	0.0028	0.0024	0.0029	0.0038	0.0033	0.0036	0.0008	0.0018	0.1227	0.9456	0.9456	H	0
506	L	0.0044	0.0011	0.0028	0.0034	0.0026	0.0041	0.0013	0.0037	0.0032	0.7216	0.2278	0.0024	0.0028	0.0023	0.0028	0.0038	0.0033	0.0041	0.0008	0.0018	0.289	0.7216	0.7216	L	0
507	L	0.0064	0.0011	0.0028	0.0034	0.0058	0.0068	0.0013	0.0037	0.0032	0.8843	0.0541	0.0024	0.0028	0.0023	0.0028	0.0068	0.0033	0.0041	0.0008	0.0018	0.2028	0.8843	0.8843	L	0
508	L	0.0047	0.0012	0.0031	0.0037	0.2155	0.0044	0.0018	0.0032	0.0035	0.0494	0.0764	0.0027	0.003	0.0025	0.0032	0.0042	0.0035	0.0039	0.0011	0.6093	0.4207	0.0494	0.6093	Y	0.5599
509	H	0.0043	0.0011	0.0029	0.0035	0.0022	0.0041	0.9448	0.0029	0.0033	0.005	0.0013	0.0025	0.0028	0.0032	0.0029	0.0038	0.0033	0.0036	0.0008	0.0018	0.1242	0.9448	0.9448	H	0
510	M	0.0043	0.0011	0.0029	0.0035	0.0022	0.0041	0.0014	0.0029	0.0033	0.005	0.9455	0.0025	0.0028	0.0024	0.0029	0.0038	0.0033	0.0036	0.0008	0.0018	0.1228	0.9455	0.9455	M	0
511	D	0.0241	0.0019	0.1135	0.7701	0.0038	0.0072	0.0053	0.0051	0.0081	0.0087	0.0023	0.0083	0.005	0.005	0.0054	0.0094	0.006	0.0064	0.0013	0.0031	0.3371	0.1135	0.7701	E	0.6566
512	A	0.345	0.0015	0.0032	0.0039	0.0026	0.0051	0.0015	0.0066	0.0036	0.0603	0.4266	0.0027	0.0031	0.0026	0.0038	0.0347	0.0828	0.0075	0.0009	0.002	0.502	0.345	0.4266	M	0.0816
513	G	0.005	0.0012	0.0099	0.0038	0.0023	0.9289	0.0015	0.003	0.0037	0.0051	0.0014	0.0032	0.0031	0.0025	0.0032	0.0125	0.0035	0.0037	0.0008	0.0019	0.15	0.9289	0.9289	G	0
514	M	0.0043	0.0011	0.0029	0.0035	0.0022	0.0041	0.0014	0.0029	0.0033	0.005	0.9455	0.0025	0.0028	0.0024	0.0029	0.0038	0.0033	0.0036	0.0008	0.0018	0.1228	0.9455	0.9455	M	0
515	F	0.0043	0.0011	0.0029	0.0035	0.8843	0.0041	0.0014	0.0029	0.0033	0.0083	0.06	0.0025	0.0028	0.0024	0.0029	0.0039	0.0033	0.0036	0.0008	0.0018	0.1979	0.8843	0.8843	F	0
516	R	0.0043	0.0011	0.0029	0.0035	0.0022	0.0041	0.0014	0.0029	0.0033	0.005	0.0013	0.0025	0.0028	0.0024	0.947	0.0038	0.0033	0.0036	0.0008	0.0018	0.1197	0.947	0.947	R	0
517	E	0.0096	0.0011	0.0047	0.8577	0.0022	0.0042	0.0014	0.0117	0.0313	0.0052	0.0014	0.0027	0.003	0.0088	0.018	0.0042	0.0255	0.0047	0.0008	0.0018	0.253	0.8577	0.8577	E	0
518	V	0.0044	0.0011	0.0029	0.0035	0.0023	0.0041	0.0014	0.0325	0.0033	0.0076	0.0061	0.0025	0.0029	0.0024	0.0029	0.0039	0.0033	0.9101	0.0008	0.0018	0.1735	0.9101	0.9101	V	0
519	V	0.0858	0.0012	0.018	0.0145	0.0024	0.0045	0.0291	0.0102	0.0111	0.0267	0.0015	0.0029	0.0031	0.0074	0.5716	0.0045	0.0064	0.1951	0.0017	0.002	0.4968	0.1951	0.5716	R	0.3765
520	V	0.0045	0.0012	0.0031	0.0037	0.0023	0.0043	0.0015	0.0031	0.0035	0.0053	0.0014	0.0026	0.003	0.0025	0.0031	0.0041	0.0035	0.9446	0.0008	0.0019	0.1243	0.9446	0.9446	V	0
521	A	0.3903	0	0	0	0	0.0001	0	0	0	0	0	0	0	0	0	0.6094	0	0	0	0	0.2243	0.3903	0.6094	S	0.2191
