# Supplementary figures and images for: Influence of mutations at different distances from the active center on the activity and stability of laccase 13B22
Source: Bioresour Bioprocess. 2025 May 27;12(1):47. doi: 10.1186/s40643-025-00893-6 (PMC12116972; doi:10.1186/s40643-025-00893-6)

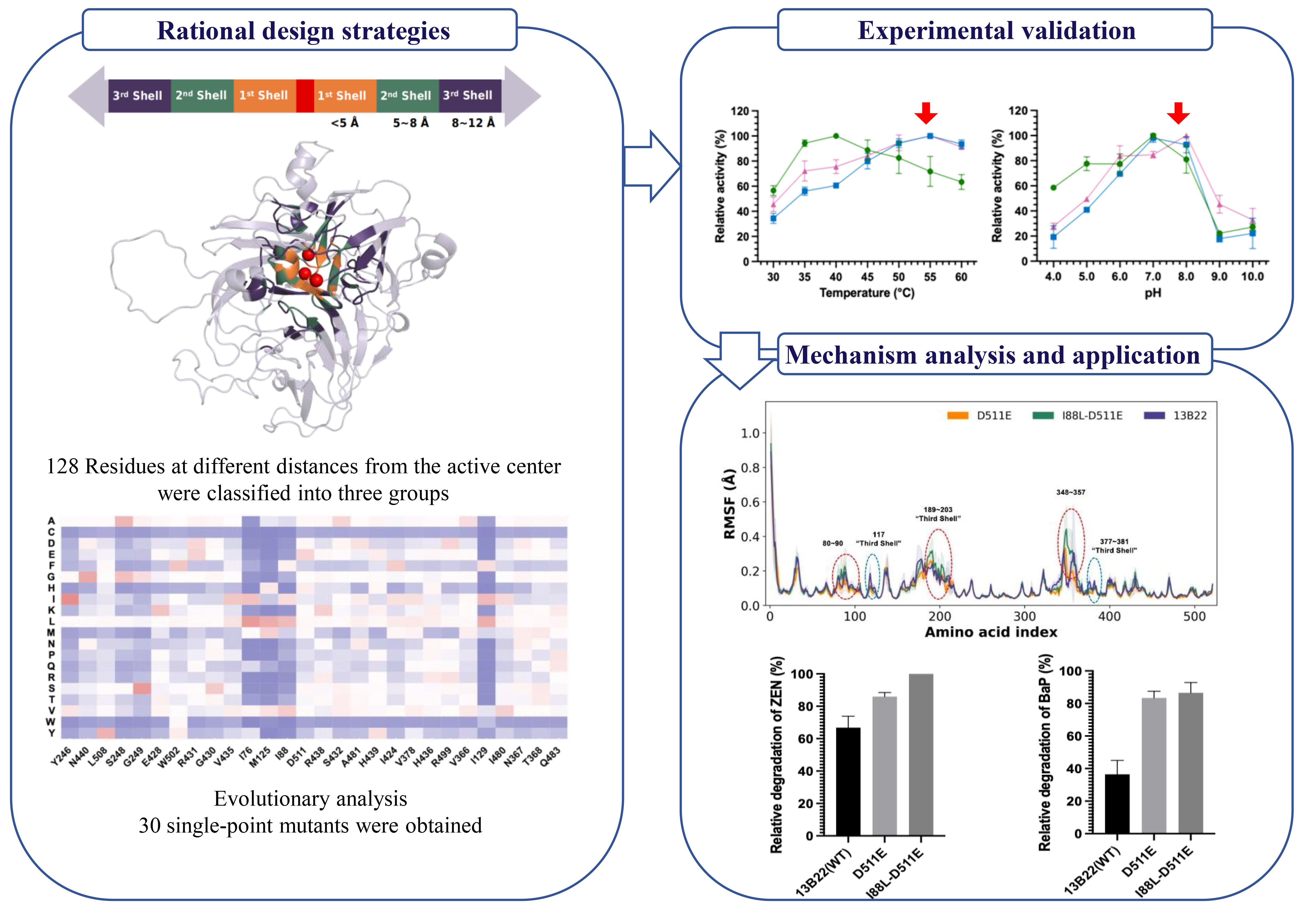

Supplement: Supplementary file 3 — Supplementary Material 3 [file 40643_2025_893_MOESM3_ESM.jpg]
